# Distinct mechanisms of planar polarization by the core and Fat-Dachsous planar polarity pathways in the *Drosophila* wing

**DOI:** 10.1016/j.celrep.2022.111419

**Published:** 2022-09-27

**Authors:** Amy Brittle, Samantha J. Warrington, Helen Strutt, Elizabeth Manning, Su Ee Tan, David Strutt

**Affiliations:** 1 School of Biosciences, University of Sheffield, Western Bank, Sheffield S10 2TN, UK

**Keywords:** planar polarity, planar cell polarity, PCP, Fat, Dachsous, Frizzled

## Abstract

Planar polarity describes the coordinated polarization of cells within a tissue plane, and in animals can be determined by the “core” or Fat-Dachsous pathways. Current models for planar polarity establishment involve two components: tissue-level “global” cues that determine the overall axis of polarity and cell-level feedback-mediated cellular polarity amplification. Here, we investigate the contributions of global cues versus cellular feedback amplification in the core and Fat-Dachsous pathways during *Drosophila* pupal wing development. We present evidence that these pathways generate planar polarity via distinct mechanisms. Core pathway function is consistent with strong feedback capable of self-organizing cell polarity, which can then be aligned with the tissue axis via weak or transient global cues. Conversely, generation of cell polarity by the Ft-Ds pathway depends on strong global cues in the form of graded patterns of gene expression, which can then be amplified by weak feedback mechanisms.

## Highlights

• The core and Fat-Dachsous planar polarity pathways function via distinct mechanisms
• The core can self-organize planar polarity and be oriented by weak upstream cues
• Fat-Dachsous are oriented by strong gradient cues but show poor self-organization

## Graphical abstract

**Figure:**
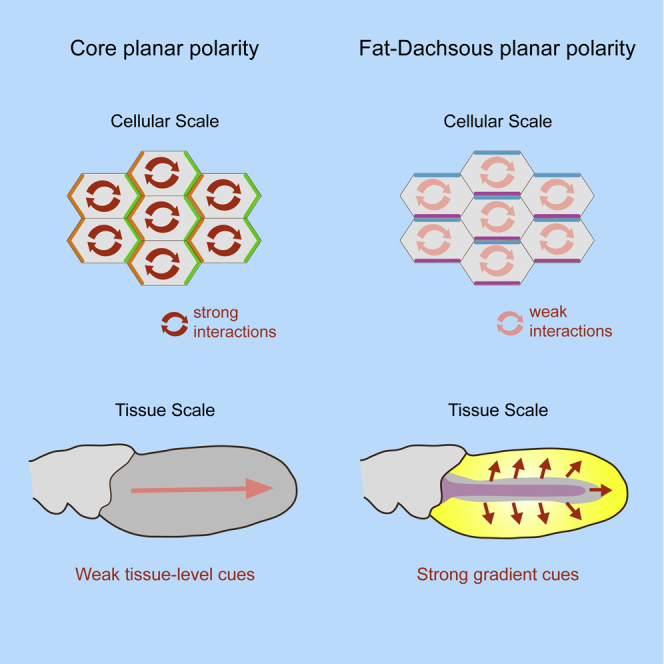

## Teaser

Brittle et al. investigate how two different pathways generate planar polarity. Behavior of the Frizzled-dependent “core” pathway suggests the ability to self-organize planar polarity that can be oriented by weak tissue-level direction cues. Conversely, the Fat-Dachsous pathway shows only weak self-organizing properties and dependence on strong tissue-level gradient cues.

## Introduction

**Figure 1:**
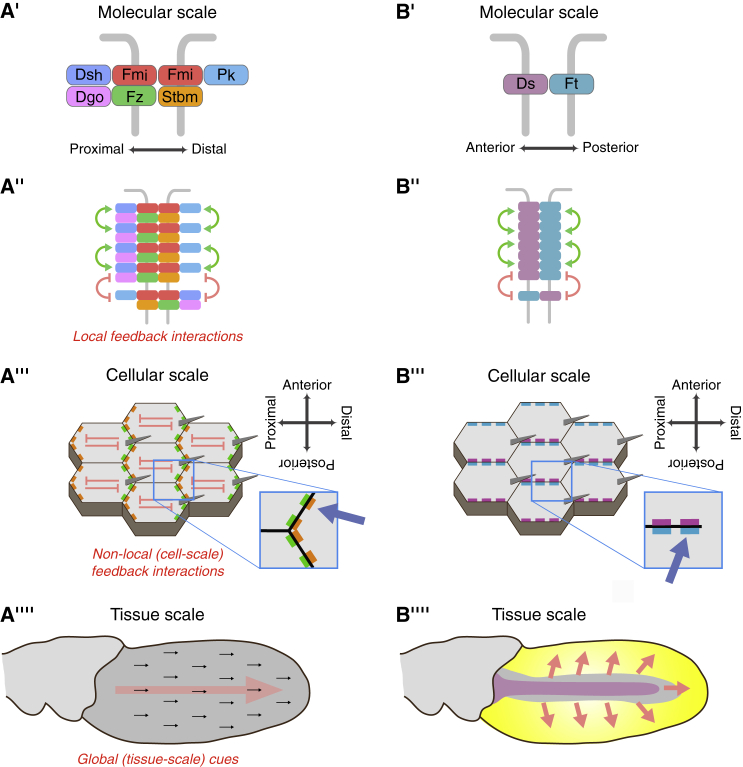
Organization of the core and Ft-Ds planar polarity pathways in the pupal wing (A′) The core proteins form asymmetric intercellular protein complexes at apicolateral cell junctions, consisting of a homodimer of the cadherin Flamingo (Fmi, red, also known as Starry Night) associated with the sevenpass transmembrane protein Frizzled (Fz, green) and the cytoplasmic proteins Dishevelled (Dsh, blue) and Diego (Dgo, magenta) on distal junctions, and the fourpass transmembrane protein Strabismus (Stbm, orange, also known as Van Gogh) and the cytoplasmic protein Prickle (Pk, cyan) on proximal junctions. (A″) Core protein complexes form local clusters of complexes of the same orientation (puncta) through the action of local feedback interactions. These might be positive interactions stabilizing complexes of the same orientation (green arrows) or negative interactions destabilizing complexes of opposite orientation (red symbols). (A‴) Core protein complexes are segregated to opposite cell edges, where they are concentrated in puncta (blue arrow, inset). This segregation may be promoted by non-local (cell-scale) inhibitory interactions (red). Trichomes (grey) emerge from distal cell edges due to core protein localization. (A⁗) In the wild-type pupal wing blade (dark grey), the overall direction of core polarity and trichome orientation is distal (black arrows). This is believed to result from weak global cue(s) (red arrow). Early in pupal wing development global cues specify a radial polarity pattern which from ∼16 h onward is re-oriented onto the proximodistal axis (Aigouy et al., 2010). (B′) Ft (teal) and Ds (purple) form *trans*-heterodimers between neighboring cells. (B″) Ft and Ds also cluster in cell junctions, which may result from feedback interactions (green and red symbols). (B‴) Ft-Ds complexes are localized to opposite cell edges, where they are concentrated in puncta (blue arrow, inset). In the mid-posterior half of the wild-type pupal wing, Ft is at medial (anterior) cell edges (upward in cartoon), whereas Ds is at lateral (posterior) cell edges. Trichomes emerge from distal cell edges independently of Ft-Ds localization. (B⁗) Ft-Ds polarity is radially oriented toward the wing margin (red arrows) with Ft medial (toward centre of wing) and Ds lateral (toward margin). This pattern thought to result from opposing gradients/boundaries of Ds (purple) and Fj (yellow) expression, with Fj high at the wing margin and Ds high medially.

Planar polarity is the polarization of cells within a tissue plane and is needed for the correct formation and function of many tissues (Goodrich and Strutt, 2011; Butler and Wallingford, 2017; Davey and Moens, 2017). In animals, planar polarity is often established by the “core” and Fat-Dachsous (Ft-Ds) pathways. Both control polarized cell behaviors through the asymmetric distribution of specific proteins to opposite cell edges (Figure 1) (reviewed in Aw and Devenport, 2017; Lawrence and Casal, 2018). For both pathways asymmetric protein localization is thought to occur through a combination of tissue-level “global” cues determining the axis of polarization and feedback mechanisms amplifying cellular asymmetry. However, the relative contributions of global cues and feedback in each pathway has not been systematically investigated.

The core pathway consists of six proteins that assemble into intercellular complexes (Figure 1A′) in punctate domains at cell junctions in the *Drosophila* wing. In these “puncta” core protein complexes are highly aligned in the same orientation (Figure 1A″), consistent with the existence of feedback interactions locally sorting complexes (Strutt et al., 2011; Cho et al., 2015). Moreover, the core pathway can generate *de novo* swirling patterns of planar polarity in the pupal wing following pathway activation (Strutt and Strutt, 2002, 2007). Similar spontaneous emergence of locally coordinated planar polarization is also observed for mammalian core proteins in primary cultures of mouse tracheal and skin cells (Aw et al., 2016; Vladar et al., 2012). This “self-organization” of planar polarity implies the presence of “non-local” feedback interactions that act at the cellular level to promote protein segregation to opposite cell edges (Figure 1A‴). In normal development these feedbacks are believed to be biased by global cues that align polarity on the tissue axis (Figure 1A⁗). The identity of the global cues in the pupal wing is unclear but may include polarized intracellular transport, morphogen gradients altering core protein activity, and polarized cell rearrangements (reviewed in Aw and Devenport, 2017).

Key proteins in the Ft-Ds pathway are the cadherins Ft and Ds and the kinase Four-jointed (Fj). Unlike classical cadherins, Ft and Ds do not bind homophilically in *trans* to themselves, but instead bind heterophilically at cell junctions to form *trans*-heterodimers (Figure 1B′) (Ma et al., 2003; Matakatsu and Blair, 2004). Four-jointed acts in the Golgi to phosphorylate Ft and Ds extracellularly on their cadherin domains, modifying their *trans*-binding affinities (Brittle et al., 2010; Simon et al., 2010). The global cue results from tissue-level gradients and boundaries of Fj and Ds expression (Figure 1B⁗): differing local levels of Fj and Ds modulate Ft-Ds binding affinities and promote the asymmetric distribution of Ft-Ds complexes to opposite cell edges (Figure 1B‴) (Ambegaonkar et al., 2012; Bosveld et al., 2012; Brittle et al., 2012; Hale et al., 2015).

Computational modelling suggests gradients and boundaries of Fj and Ds expression are sufficient to generate cellular asymmetry without amplification (e.g., Jolly et al., 2014; Hale et al., 2015; Wortman et al., 2017). However, it is unclear whether the shallow *in vivo* expression gradients explain the observed levels of cellular asymmetry, and hence it has been speculated that feedback amplification also contributes (Figure 1B″) (Ambegaonkar et al., 2012; Brittle et al., 2012; Mani et al., 2013; Hale et al., 2015). Notably, work with the mammalian homologs Fat4 and Dchs1 in cultured cells supports the presence of feedback mechanisms leading to local alignment of complexes of the same orientation (Loza et al., 2017).

Here, we investigate mechanisms of planar polarization in the core and Ft-Ds pathways in the *Drosophila* wing. We assess the relative contributions of tissue-level global signals and feedback mechanisms by assaying polarization at three levels: individual clusters of proteins, single cells, and locally in the tissue (Figure 1). Our results support these pathways generating planar polarity via distinct mechanisms: the core pathway shows evidence of strong feedback-mediated self-organization biased by weak global cues, whereas Ft-Ds show dependence on strong global cues to generate cell polarity which can be amplified by weaker feedbacks.

## Results

### Increased asymmetry of core proteins and Ft-Ds in puncta

The presence of punctate clusters of core proteins consisting of stable complexes in a common orientation (Strutt et al., 2011, 2016) provides evidence for sorting via local feedback interactions (Figure 1A″). Ft and Ds also show punctate localization (Ma et al., 2003; Hale et al., 2015) and we have shown previously using fluorescence recovery after photobleaching (FRAP) that Ft and Ds are more stable in these regions (Hale et al., 2015). To seek evidence for local feedback in the Ft-Ds system we examined Ft-Ds puncta to determine if they show high alignment of Ft-Ds heterodimers.

**Figure 2:**
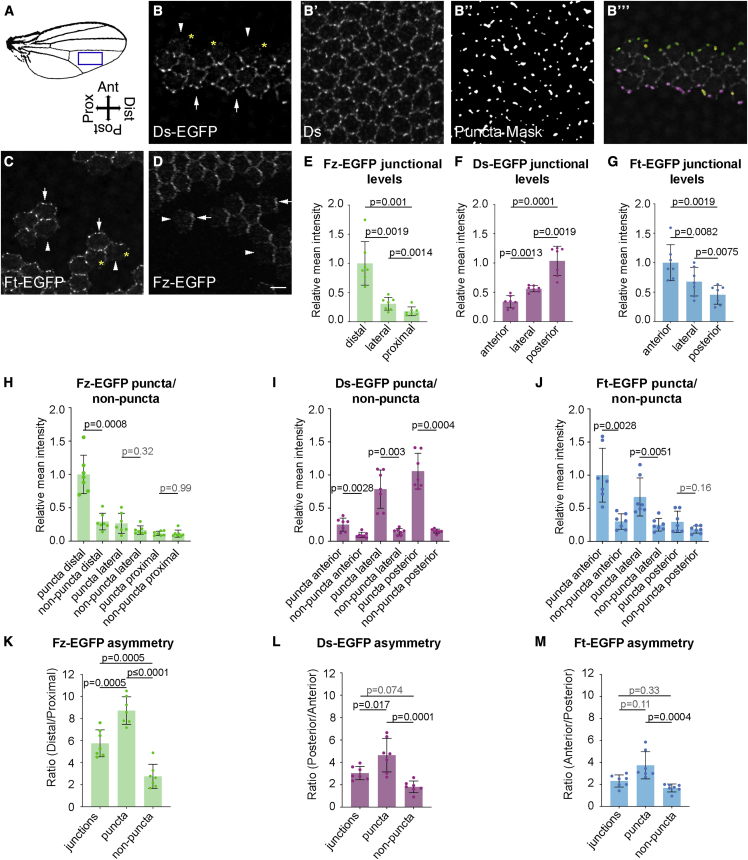
Puncta are sites of aligned asymmetric complexes in both the core and Ft-Ds pathways (A) Diagram of adult wing indicating region analyzed below vein 4 (blue box). (B–D) Images of *ds-EGFP*, *ft-EGFP*, and *fz-EGFP* twin-clones next to tissue with untagged protein, revealing asymmetric cellular localizations on clone boundaries. Arrows point to high EGFP levels: Ds-EGFP on posterior junctions (B), Ft-EGFP anterior (C), Fz-EGFP distal (D). Arrowheads point to low EGFP junctions. Asterisks indicate weak EGFP puncta on anterior ((B) Ds-EGFP) and posterior ((C) Ft-EGFP) junctions. All images from 28 h pupal wings below vein 4. Distal right and posterior down here and subsequently. Scale bar, 5 μm. (B′–B‴) Selection of puncta for analysis. Ds immunolabeling (B′) was used to select puncta (B″, see STAR Methods) on clone boundaries between *ds-EGFP* and untagged (*ds*^*+*^) tissue which were classified according to boundary orientation. Here, puncta are false-colored depending on boundary orientation—posterior (purple), lateral (yellow), and anterior (green) (B‴). (E–G) Normalized mean intensity of EGFP on cell junctions at clone boundaries for *fz-EGFP* (E), *ds-EGFP* (F), and *ft-EGFP* (G). (H–J) Normalized mean intensity of EGFP in puncta and non-puncta. (K–M) Mean asymmetry of EGFP in junctions, puncta, and non-puncta. All means were compared with repeated measure ANOVA analysis ((E–G) and (K–M) are ANOVA with Tukey’s multiple comparisons test and (H–J) are ANOVA with Sidak’s multiple comparisons test to compare between selected columns), p values as indicated. Error bars are standard deviation. n = 7 wings per genotype. See also Figure S1.

To study protein localization, we used the core protein Frizzled (Fz) and Ft and Ds tagged with EGFP at their endogenous loci (Brittle et al., 2012; Hale et al., 2015; Strutt et al., 2016). To enable consistent comparisons between the two pathways, images were taken in the same region below vein 4 (Figure 2A) in the 28 h APF (after puparium formation) wing, a stage when both pathways exhibit clear planar polarization and cell rearrangements are minimal (Strutt, 2001; Aigouy et al., 2010; Merkel et al., 2014). Measurements were made on the boundaries of “twin-clones”, where tissue expressing tagged protein was juxtaposed to tissue expressing untagged protein (Figures 2B–2D). In this way we can determine the amount of a protein specifically localized on one side of a junction. We measured mean intensity of EGFP as a proxy for Fz, Ft, and Ds protein levels, in whole junctions, within puncta and within non-puncta regions. Puncta were identified using thresholding on tissue immunolabeled for the core protein Flamingo (Fmi) for Fz-containing puncta, and for Ds protein for Ft-Ds puncta (Figures 2B′ and 2B″; see STAR Methods) (aee also Strutt et al., 2016). Individual puncta were also classified by the orientation of the boundary on which they were localized (Figure 2B‴).

Measurement of mean Fz-EGFP intensity on whole junctions on twin-clone boundaries confirmed enrichment on distal cell junctions, with intermediate levels on lateral junctions (Figures 2D and 2E) (Strutt et al., 2011; Strutt et al., 2016). Furthermore, in distal junctions Fz-EGFP was significantly enriched in puncta versus non-puncta, unlike on lateral and proximal junctions (Figure 2H). Based on the ratio of mean intensities, proximodistal asymmetry was overall highest in puncta compared with either whole junctions or non-puncta (Figure 2K).

In the pupal wing, Ft-Ds cellular asymmetry radiates out toward the wing margin in a broadly anteroposterior orientation, following the gradients of Ds and Fj expression (Figures 1B‴ and 1B⁗) (Merkel et al., 2014). We again measured EGFP intensity on twin-clone boundaries. Ds-EGFP intensity was stronger on posterior than anterior junctions and intermediate on lateral junctions (Figures 2B and 2F). As expected Ft polarity was the opposite of Ds (Figures 2C and 2G).

Notably, asymmetry of Ds-EGFP and Ft-EGFP within puncta is higher compared with non-puncta and whole junctions (Figures 2I, 2J, 2L, and 2M), although the degree of asymmetry is about half that seen for Fz-EGFP. We also confirmed at an earlier developmental time point in the wing disc that Ft-EGFP and Ds-EGFP are more strongly polarized in puncta (Figures S1A–S1F), although again the asymmetry is weaker than for Fz-EGFP. In conclusion, Ft-Ds complexes show greater asymmetry within puncta than within non-puncta (albeit weaker than seen for the core pathway) supporting the presence of local sorting.

### Stable Ft-Ds complexes are present in both orientations on anteroposterior cell junctions

When examining Ds-EGFP on clone boundaries, in addition to bright puncta on posterior cell edges, we also see weaker but distinct Ds-EGFP puncta on anterior edges (asterisks Figure 2B). Similarly, we can see some punctate Ft-EGFP on posterior junctions (Figure 2C). Conversely, discrete puncta of proximal Fz-EGFP (Figure 2D) are not easily evident. We also imaged Ds-EGFP and Fz-EGFP at high resolution in the wing disc and again saw Ds-EGFP but not Fz-EGFP (Figures S1G and S1H) on proximal junctions in discrete puncta (note both Ft-Ds and core polarity are oriented proximodistally in the wing disc). Hence, for Ft and Ds there is a “wrongly” oriented punctate population at cell junctions.

This raises two questions. First, is this oppositely oriented Ft-Ds stably bound as heterodimers between neighboring cells? Second, if there are stable Ft-Ds heterodimers of the “wrong” orientation, do they form distinct puncta of their own or are they forming mixed puncta with heterodimers in both orientations?

**Figure 3:**
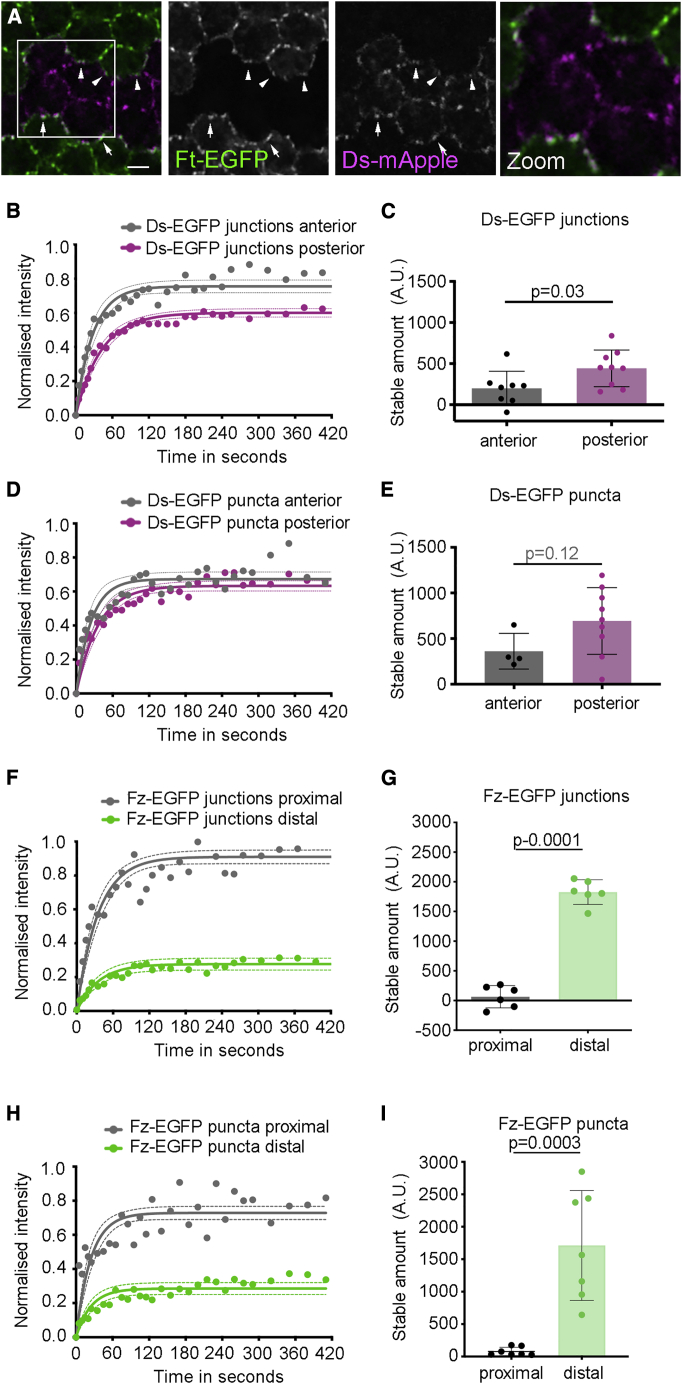
Ds is stable on both anterior and posterior cell edges (A) Image of *ft-EGFP/ds-mApple* twin-clone in 28 h pupal wing. Posterior Ds-mApple (magenta) and anterior Ft-EGFP (green) co-localize in puncta (arrows). Weaker punctate posterior Ft-EGFP and anterior Ds-mApple also co-localize (arrowheads). In zoomed image (right) separation of Ft-EGFP and Ds-mApple can be seen, consistent with them being localized on opposite sides of cell junctions. (B and C) FRAP analysis of Ds-EGFP on whole anterior and posterior junctions. (B) Recovery of Ds-EGFP intensity after photobleaching. (C) Mean intensity of the stable amount of Ds-EGFP (A.U.), calculated by multiplying the stable fraction by the total fluorescence (unpaired t test p = 0.03, anterior n = 7 wings, posterior n = 8 wings). Note, ratio of Ds-EGFP on posterior versus anterior junctions in live imaging is lower than in fixed samples (Figure 2F) due to differences in sample preparation. (D and E) FRAP analysis of punctate Ds-EGFP. (D) Recovery of Ds-EGFP intensity. (E) Mean intensity of the stable amount of Ds-EGFP in puncta (unpaired t test p = 0.12, anterior n = 4 wings, posterior n = 9 wings). (F and G) FRAP analysis of Fz-EGFP on whole proximal and distal junctions. (F) Recovery of Fz-EGFP intensity after photobleaching. (G) Mean intensity of the stable amount of Fz-EGFP (unpaired t test, p = 0.0001, proximal n = 7 wings, distal n = 7 wings). (H and I) FRAP analysis of Fz-EGFP puncta on proximal and distal junctions. Puncta were identified by mCherry-Diego localization. (H) Recovery of Fz-EGFP intensity. (I) Mean intensity of the stable amount of Fz-EGFP in puncta (unpaired t test, p = 0.0003, proximal n = 7 wings, distal n = 7 wings). Table S1 contains the summarized data and 95% confidence intervals for all the FRAP experiments. See also Table S1.

To address the first question, we asked whether punctate anterior Ds co-localized with Ft in the apposed posterior junctions. We generated twin-clones expressing Ds-mApple next to Ft-EGFP. Punctate Ds-mApple on anterior clone boundaries corresponded with Ft-EGFP puncta in the neighboring cell (arrowheads Figure 3A) supporting heterodimeric Ft-Ds binding. The localization does not completely overlap, consistent with Ft and Ds being on opposite sides of junctions.

We previously used FRAP to show that Ft and Ds junctional stability is lost in the absence of each other, implying stability depends on heterodimer formation (Hale et al., 2015). FRAP carried out on whole junctions from twin-clone-bearing wings revealed that Ds-EGFP on anterior cell edges shows a stable fraction of ∼25% (i.e., ∼75% recovery after photobleaching) compared with ∼40% on posterior cell edges (Figure 3B; Table S1). Considering total levels of Ds-EGFP on the respective junctions, this reveals that there is a stable population of Ds-EGFP on anterior junctions almost half the size of that on posterior junctions (Figure 3C). Moreover, when FRAP was carried out on punctate Ds-EGFP on posterior and anterior junctions, we again found significant stable fractions and amounts (Figures 3D and 3E; Table S1).

Fz-EGFP does not appear on the proximal side of junctions in obvious puncta (Figure 2D). To test for the stability of Fz-EGFP on proximal junctions we generated twin-clones in a background containing mCherry-Diego (a core protein that co-localizes with Fz and Fmi; Figure 1A′) (Feiguin et al., 2001; Das et al., 2004). FRAP detected a negligible stable population of Fz-EGFP on proximal cell edges (Figures 3F and 3G). We then used mCherry-Diego to identify sites of core protein puncta and carried out FRAP on Fz-EGFP. Again, negligible levels of stable Fz-EGFP were detected in proximal puncta (Figures 3H and 3I).

Overall, we find a stable punctate population of Ds on both anterior and posterior cell junctions, supporting the presence of stable Ft-Ds heterodimers of both orientations. This is not true in the core system where there is negligible stable Fz on proximal junctions.

### Individual Ft-Ds puncta contain stable complexes in both orientations

**Figure 4:**
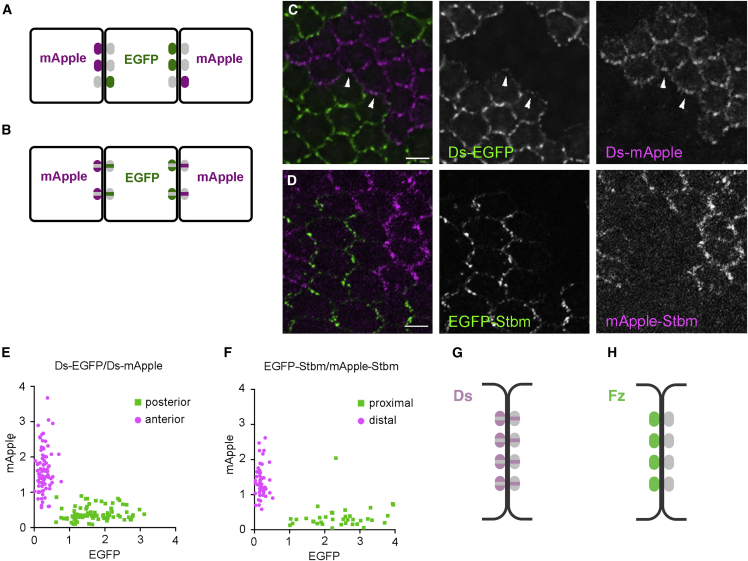
Ft-Ds puncta are mixed with complexes in both orientations (A and B) Schematic of EGFP/mApple twin-clone experiments to examine puncta composition. Cells express a protein tagged with EGFP (green) or mApple (magenta), and puncta are examined at clone boundaries between cells (grey indicates unlabeled *trans*-binding partners). (A) A situation where individual puncta are strongly polarized, but some puncta are polarized in the opposite orientation. (B) Mixed puncta with complexes primarily in one orientation but with a proportion in the opposite orientation. (C and D) Images of twin-clone tissue in a 28 h pupal wing. (C) *ds-EGFP*/*ds-mApple* twin-clones. Arrowheads indicate punctate anterior Ds-EGFP co-localizing with posterior Ds-mApple in neighboring junctions. (D) *EGFP-stbm/mApple-stbm* twin-clones. Distal EGFP-Stbm does not form distinct puncta. Scale bars, 5 μm. (E and F) Scatter plots of EGFP versus mApple intensity in puncta on twin-clone boundaries. Puncta from multiple wings pooled. (E) Puncta between *ds-EGFP/ds-mApple* tissue divided into those on the anterior (magenta) or posterior (green) boundary relative to EGFP (n = 6 wings). (F) Puncta between *EGFP-stbm/mApple-stbm* tissue divided into those on proximal (green) or distal (magenta) boundaries (n = 5 wings). In both cases, puncta groups are either EGFP > mApple or mApple > EGFP, consistent with the model in (B) where puncta on a boundary of a particular orientation show the same overall polarity. (G and H) Diagrams illustrating deduced distributions of stable complexes in puncta. (G) Ft-Ds puncta have stable Ds on both posterior and anterior sides of junctions, consistent with puncta being mixed and stable Ft-Ds heterodimers being present in both orientations. (H) Core pathway puncta only have stable Fz on distal cell edges, consistent with stable core pathway complexes being present in only one orientation. See also Figure S2.

As noted above, it is possible that individual Ft-Ds puncta are well sorted, but that puncta of both orientations are present (Figure 4A). Alternatively, individual puncta may be mixed (Figure 4B). To investigate this, twin-clones juxtaposing Ds-EGFP versus Ds-mApple were generated (Figure 4C) and puncta identified by Ds immunolabeling (Figure S2). Notably, we observed weak punctate anterior Ds-EGFP co-localizing with Ds-mApple in neighboring posterior junctions (arrowheads Figure 4C), consistent with the presence of mixed puncta.

To analyze further, we selected puncta lying either on anterior or posterior junctions relative to the Ds-EGFP clone tissue and plotted the fluorescent intensities of EGFP (in the clone tissue) and mApple (in the apposed twin-clone) on a scatter graph. As expected, if the Ds signal is always higher on posterior cell edges, the points clustered into two populations: puncta on posterior Ds-EGFP clone edges (green dots) with higher Ds-EGFP/lower Ds-mApple, and puncta on anterior Ds-EGFP clone edges (magenta dots) with higher Ds-mApple/lower Ds-EGFP (Figure 4E). If there was a subpopulation of oppositely oriented puncta with higher Ds anteriorly, we would expect to see green dots localized within the magenta cloud and vice versa. We conclude that Ft-Ds puncta are mixed but with a strong bias toward having more Ds on posterior edges.

We carried out a similar experiment to examine core pathway puncta. As we do not have Fz tagged with a red fluorescent protein, we instead generated EGFP-(Strabismus) Stbm/mApple-Stbm-expressing twin-clones (Figure 4D) for the core protein Stbm which localizes on proximal cell edges (Figure 1A) (Bastock et al., 2003; Strutt et al., 2016) and identified puncta on clone boundaries, classifying them as proximal or distal to the EGFP clone. Again we found that puncta were overall polarized in the same orientation (Figure 4F).

In conclusion, for both Ft-Ds and the core pathway, individual puncta have a common orientation. However, Ft-Ds puncta contain detectable populations of Ds on both anterior and posterior cell edges that FRAP reveals to be in stable complexes (Figure 3E). This suggests that Ft-Ds puncta contain a mixture of stable Ft-Ds heterodimers in both orientations (Figure 4G). Conversely, in the core system, puncta do not contain a significant population of stable Fz-containing complexes of the wrong orientation (Figure 4H).

### *De novo* self-organization of planar polarity by the core pathway but not the Ft-Ds pathway

The ability of the core pathway to self-organize to give locally coordinated swirling patterns of planar polarity has been reported previously (see introduction), implying the presence of feedback mechanisms allowing complexes to spontaneously segregate to opposite cell edges (Figure 1A‴). In the pupal wing, strong swirling patterns are seen when core pathway activity is induced *de novo* after ∼16 h APF (Strutt and Strutt, 2002, 2007), suggesting that, by this stage of development, global cues are either absent or too weak to specify consistent proximodistal polarity.

**Figure 5:**
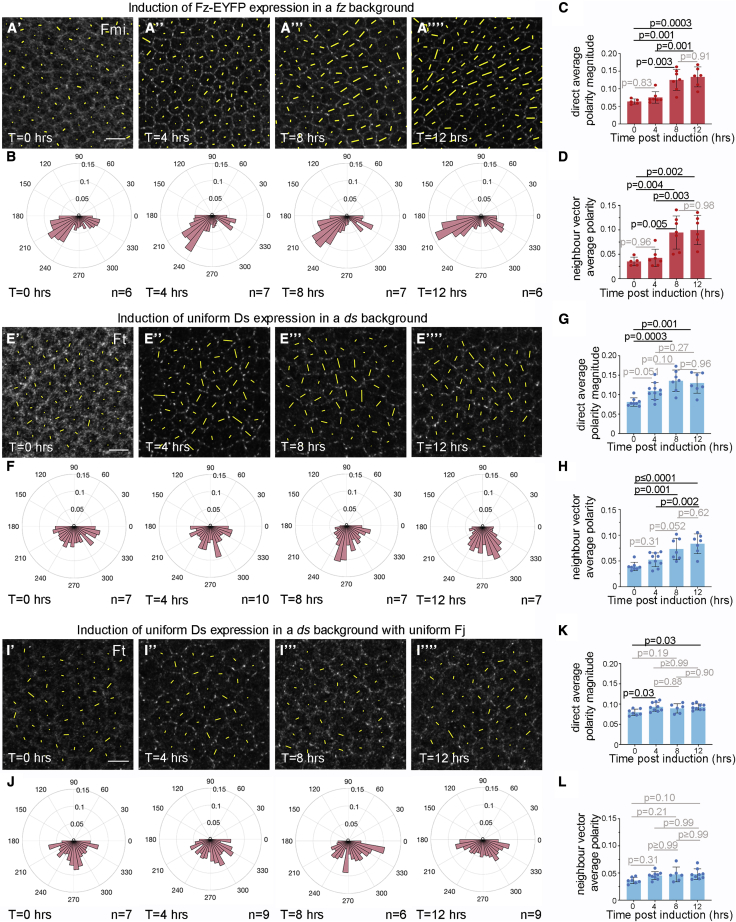
The core pathway but not the Ft-Ds pathway *de novo* self-organizes coordinated cellular polarity (A, E, and I) Images of 28 h pupal wings showing effects of protein expression for the indicated periods, immunolabeled to show normalized protein intensity (greyscale) and cell polarity marked by yellow lines. (A) Fz-EYFP expression in an *fz* background, immunolabeled for Fmi. (E) Ds expression in a *ds* background, immunolabeled for Ft. (I) Ds expression in a *ds* background with uniform Fj expression, immunolabeled for Ft. Scale bars, 5 μm. (B, F, and J) Circular weighted histograms, representing polarity angles of individual cells for different lengths of protein expression. Data pooled from multiple wings (n = number of wings). (B) Fz-EYFP expression in an *fz* background. (F) Ds expression in a *ds* background. (J) Ds expression in a *ds* background with uniform Fj expression. (C, G, and K) Average cell polarity (direct average polarity magnitude) versus length of expression. (C) Fmi polarity upon Fz-EYFP expression in an *fz* background. (G) Ft polarity upon Ds expression in a *ds* background. (K) Ft polarity upon Ds expression in a *ds* background with uniform Fj expression. Note, slight increase as Ft changes from a diffuse membrane localization to a punctate distribution between 0 and 4 h, with no increase thereafter. We attribute the small increase to a random punctate distribution having a less uniform membrane localization than a diffuse distribution. (D, H, and L) Locally coordinated polarity magnitude (neighbor vector average polarity) versus length of expression. (D) Fmi polarity upon Fz-EYFP expression in an *fz* background. (H) Ft polarity upon Ds expression in a *ds* background. (L) Ft polarity upon Ds expression in a *ds* background with uniform Fj expression. In (L), again note no increase beyond 4 h. All means were compared with ANOVA analysis (Tukey’s multiple comparisons test to compare all columns) and p values indicated. Error bars are standard deviation. See also Figure S3.

To further test the roles of feedback mechanisms in core and Ft-Ds planar polarity we directly compared their abilities to polarize following *de novo* pathway activation in the pupal wing. As previously we turned on expression of Fz-EYFP from the *Actin5C* promoter in an *fz* background using a heat-shock (Figure S3A; see STAR Methods) (Strutt and Strutt, 2002). Fz-EYFP was expressed for varying times prior to examining polarity at 28 h APF. We confirmed that Fz-EYFP was not present without heat-shock and levels increased with longer expression (Figures S3D–S3G). Over time Fmi protein changes from a diffuse junctional localization to a punctate and polarized localization (Figures 5A and S3D′–S3G′). Quantitation showed significant increases in both individual cell polarity (Figure 5C) and locally coordinated polarity between neighboring cells (Figures 5B and 5D; see STAR Methods for details of polarity quantitation). As reported previously (Strutt and Strutt, 2002, 2007), planar polarity follows swirling patterns and below vein 4 is oriented on an intermediate axis between proximodistal and anteroposterior (Figures 5A and 5B).

To test the ability of Ft-Ds to self-organize we used similar induction experiments, turning on expression of Ds uniformly under the *armadillo* (*arm*) promoter in a *ds* background. We first confirmed that Ds expressed under the *arm* promoter throughout development polarizes normally on the anteroposterior axis (Figures S3H and S3I). When Ds expression was temporally induced in a *ds* background we again saw protein gradually accumulating at junctions (Figures S3J–S3M). Continued expression led to punctate localization of Ds and Ft (Figures 5E, S3J–S3M, and S3J′–S3M′) and the planar polarization of Ft increased over time (Figures 5E–5H). By 8–12 h induction Ft asymmetry was aligned on the anteroposterior axis (Figure 5F), in the direction of the Fj gradient cue (see Figure 1B⁗).

To test whether the global cue provided by the Fj gradient was absolutely required for coordinated Ft-Ds polarity, we then flattened the Fj gradient. Rather than looking in *fj* mutant wings, where Ft-Ds binding might be reduced (Brittle et al., 2010; Simon et al., 2010; Hale et al., 2015), we generated uniform Fj by expressing Fj under the *Actin5C* promoter in a *fj* background, so that binding would be more similar to wild-type conditions (Figures S3B and S3C). Any contribution of graded Ds expression has already been removed by expressing Ds with the *arm* promoter in a *ds* background. Turning on Ds expression again led to punctate Ft-Ds localization (Figures 5I, S3N–S3Q, and S3N′–S3Q′). There was a small increase in measured individual cell polarity from 0 to 4 h, but no significant increase thereafter (Figure 5K). We attribute the small increase between 0 and 4 h to the change in the Ft distribution from diffuse to punctate and the random puncta distributions producing an apparent axis of polarity within cells.

Consistent with the lack of a global cue, we could not detect anteroposterior oriented polarity in the absence of graded Fj (Figures 5I and 5J). Nevertheless, if the Ft-Ds pathway is capable of self-organizing polarity, we would expect over time to see increasing local coordination between cells, as seen after induction of the core pathway (Figures 5A and 5D). However, in the absence of graded Fj, we were unable to detect a significant increase in the coordination of polarity of each cell with its immediate neighbors (Figure 5L), and the polarity angles of individual cells across the region remained dispersed (Figure 5J). We conclude that, in the absence of a global cue, Ft and Ds are not able to self-organize to produce locally coordinated planar polarity.

### Ft-Ds are more weakly sorted in puncta in the absence of the Fj and Ds gradients

Our data argue against strong feedbacks acting non-locally to segregate Ft and Ds to opposite cell edges in the absence of global cues. However, our initial observation of increased Ft-Ds asymmetry within puncta suggests that there may nevertheless be a local sorting mechanism. If this were true, sorting within puncta would be expected to occur even without global cues.

**Figure 6:**
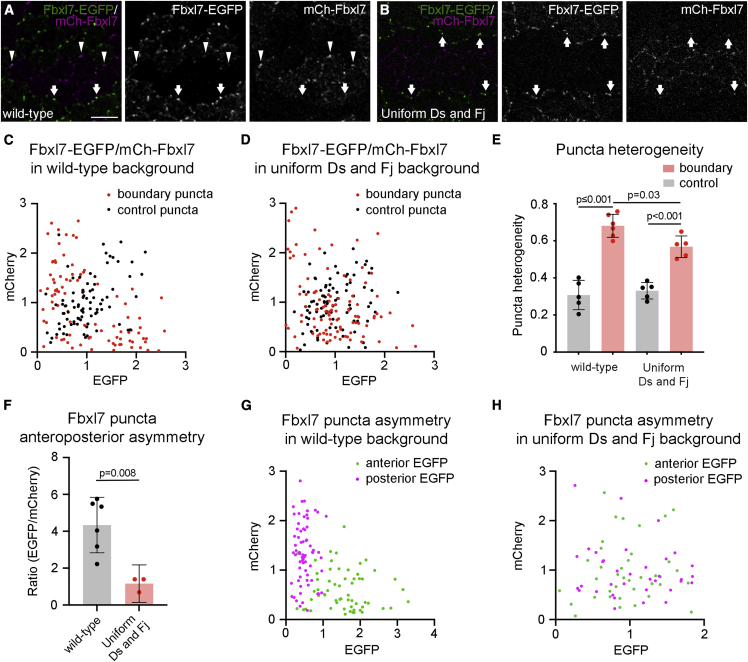
Flat gradients of Ds and Fj result in loss of anteroposterior polarity and reduced sorting of Ft-Ds in puncta (A and B) Images of 28 h pupal wings with *fbxl7-EGFP/mCherry-fbxl7* twin-clones in a wild-type background (A) or with uniform Ds and Fj expression (B). (A) Higher levels of Fbxl7-EGFP (arrows) and mCherry-Fbxl7 (arrowheads) can be seen on anterior cell junctions on clone boundaries. (B) Arrows indicate puncta on anterior and posterior junctions where Fbxl7-EGFP and mCherry-Fbxl7 co-localize. Scale bar, 5 μm. (C and D) Scatter plots of intensity of EGFP and mCherry in puncta on twin-clone boundaries (red dots) and control puncta in heterozygous tissue (black dots), in a wild-type background (C) or with uniform Ds and Fj (D). Data pooled from six (C) or five (D) wings. (E) Quantitation of mean degree of heterogeneity of Fbxl7 polarity in boundary and control puncta, in wild-type (n = 6) or uniform Ds and Fj (n = 5) wings. “0” indicates completely mixed and “1” completely unmixed (all Fbxl7 on one side of junctions). Errors bars are standard deviation. An ANOVA with Sidak’s multiple comparisons test was used to compare pairs of samples for significance. (F) Ratio of Fbxl7-EGFP/mCherry-Fbxl7 levels in puncta on twin-clone boundaries on the anterior of Fbxl7-EGFP clones in a wild-type background (n = 6 wings) showing anteroposterior polarization or with uniform Ds and Fj expression (n = 3 wings) showing no overall polarity. Error bars are standard deviation. Samples were compared using an unpaired t test. (G and H) Scatter plots of intensity of EGFP versus mCherry in individual puncta on boundaries, considering only those on the anterior (green) or posterior edges (magenta) of EGFP clones, in a wild-type background (G) or with uniform Ds and Fj (H).

To test this, we generated flies in which the Fj and Ds gradients were flattened and which harbored twin-clones expressing the Ft-binding protein Fbxl7 (Bosch et al., 2014; Rodrigues-Campos and Thompson, 2014) tagged with EGFP or mCherry. Fbxl7 was chosen as a read-out for Ft-Ds localization as we lack strains uniformly expressing tagged forms of Ds. In a wild-type background, as expected Fbxl7 is overall enriched in puncta on anterior cell edges (Figures 6A and 6F). However, in the absence of the Fj and Ds gradients this anterior enrichment is lost (Figures 6B and 6F), consistent with the loss of cell polarization seen in this condition (Figures 5I–5L).

To assess the degree of sorting of Ft-Ds heterodimers within individual puncta, we measured the intensity of EGFP and mCherry in puncta on twin-clone boundaries (boundary puncta) compared with that in control puncta not on twin-clone boundaries inside *Fbxl7-EGFP/mCherry-Fbxl7* heterozygous tissue (control puncta). Control puncta are expected to have on average a 1:1 ratio of normalized EGFP to mCherry signal. However, imaging noise and stochastic variation in the amounts of EGFP and mCherry in puncta is expected to produce some variation in ratios in control puncta (black dots, Figures 6C and 6D). In the boundary puncta in wild-type, most puncta contained predominantly either EGFP or mCherry, consistent with Ft-Ds heterodimers in puncta being overall polarized (red dots, Figure 6C). In the absence of the gradients, puncta contained more similar levels of EGFP and mCherry but nevertheless showed greater variation in the EGFP/mCherry ratio than the control puncta (some red dots lying outside the cloud of black dots, Figure 6D), suggesting that puncta are still polarized albeit more weakly.

To quantify these differences, for each condition a line was fitted to the heterozygous puncta distribution and the difference of each individual punctum from this line was calculated (see STAR Methods). A value close to 1 indicates a highly heterogenous EGFP/mCherry ratio (consistent with high sorting in boundary puncta) and a value of 0 indicates a completely mixed punctum. This analysis shows that heterogeneity within boundary puncta (i.e., degree of “sorting”) is highest in wild-type and reduced when the gradients are flattened (Figure 6E), with control puncta showing the lowest heterogeneity.

To explore further, we considered just puncta where the EGFP signal was on either anterior or posterior twin-clone boundaries (excluding those on lateral boundaries), and plotted the relative EGFP/mCherry intensities (Figures 6G and 6H). In wild-type, individual puncta of both populations correlate with higher Fbxl7 on anterior cell edges (green dots for anterior EGFP puncta show EGFP > mCherry, magenta dots for posterior EGFP puncta show mCherry > EGFP). Conversely, in the absence of the gradients, each population of puncta shows no bias toward having higher EGFP or mCherry, indicating that, although individual puncta have more Fbxl7 on one side or the other, this polarization is independent of the tissue axis. These data support weak sorting of Ft-Ds heterodimers within puncta that is uncoupled from global cues.

### Fbxl7 stabilizes Ft-Ds in puncta

It has been suggested that Ft-Ds complex sorting could be a result of reciprocal action of Fbxl7 on Ft and Ds, with Fbxl7 binding to Ft stabilizing Ft localization and destabilizing Ds by mediating its ubiquitination and removal (Rodrigues-Campos and Thompson, 2014). However, previous studies on the effects of loss and gain of Fbxl7 on Ft and Ds localization gave contradictory results (Bosch et al., 2014; Rodrigues-Campos and Thompson, 2014) and neither study tested effects on Ft and Ds stability.

**Figure 7:**
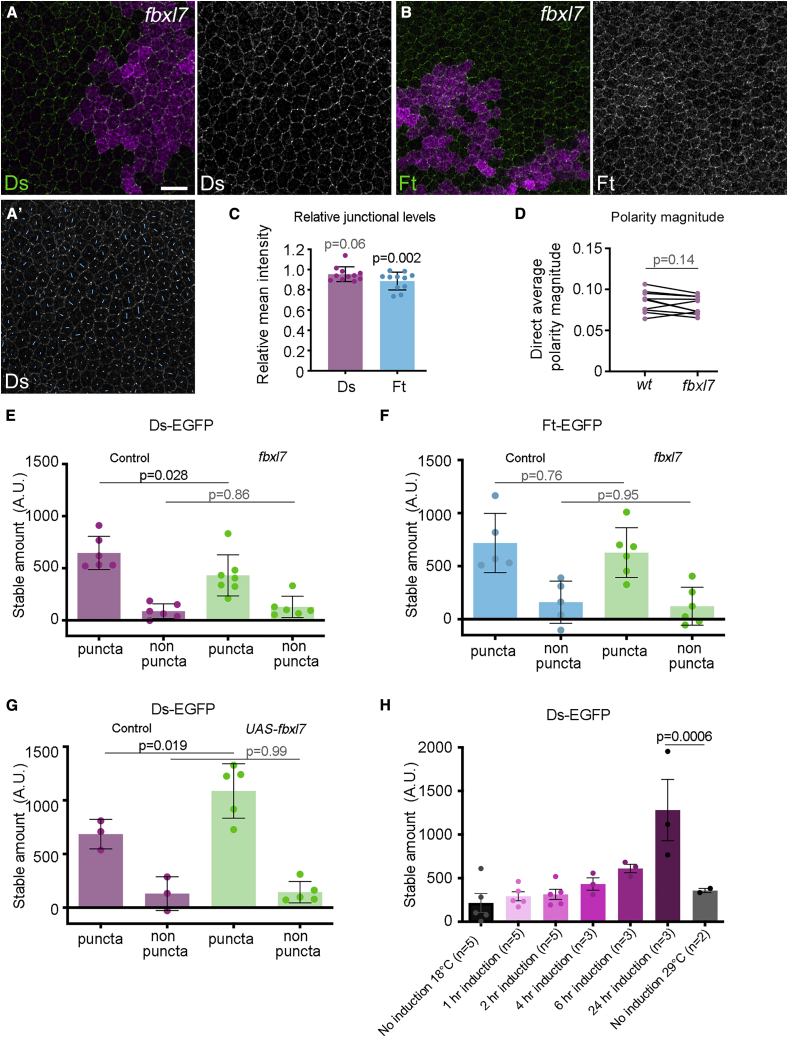
Fbxl7 is not required for Ft-Ds planar polarity but does stabilize Ft-Ds in puncta (A and B) APF pupal wings (28 h) carrying loss-of-function *fbxl7* clones below vein 4, marked by loss of β-gal immunolabeling (magenta), immunolabeled for Ds (A) or Ft (B). (A′) Ds immunolabeling with polarity nematics overlaid in blue reveals anteroposterior planar polarity inside and outside clone. Scale bar, 10 μm. (C) Mean intensity of Ds or Ft immunolabeling, shown as a ratio of intensity in *fbxl7* tissue versus wild-type tissue in the same wing. Error bars are standard deviation, n = 11 wings. One-sample t tests were used to determine if the ratios differed from 1. (D) Average cell polarity magnitude for wild-type tissue and *fbxl7* clone tissue in 28 h pupal wings immunolabeled for Ds. All clones were below vein 4. Values from the same wing are linked by black bars, n = 10. Paired t tests were used to compare values in the same wing. Polarity magnitude is relatively low as most clones analyzed lay near the wing margin. (E and F) Mean intensity of the stable amount of Ds-EGFP (E) or Ft-EGFP (F) determined using FRAP in puncta and non-puncta regions of wing discs in control tissue (purple or teal) or *fbxl7* mutant tissue (green). Error bars are standard deviation. Samples compared using ANOVA with Sidak’s multi-comparison test. Number of wings analyzed n = 6 (Ds-EGFP) or n = 5 (Ft-EGFP) for control regions, n = 7 (Ds-EGFP) or n = 6 (Ft-EGFP) for *fbxl7* mutant tissue. (G) Mean intensity of the stable amount of Ds-EGFP determined using FRAP analysis in puncta and non-puncta regions of wing discs in control tissue (purple) or *fbxl7* overexpression tissue (green) at 29°C. Error bars are standard deviation. Samples compared using ANOVA with Sidak’s multi-comparison test. Number of wings analyzed n = 3 for control regions, n = 5 for the *UAS-fbxl7* overexpression tissue. (H) Mean intensity of the stable amount of Ds-EGFP determined using FRAP analysis in puncta in wing discs. *UAS-fbxl7* overexpression is temporally induced in an *fbxl7* mutant background using *Act-GAL4*,*tub-GAL80*^*ts*^ by shifting animals from 18°C to 29°C for the time indicated. Control tissue without overexpression is shown in the left column at 18°C, and right column at 29°C. Error bars are standard deviation. Last two columns compared using ANOVA with Sidak’s multi-comparison test. Number of wings analyzed n = 2 for 29°C control regions (grey bar), n = 3 for the *UAS-fbxl7* 24 h overexpression tissue (maroon). See also Figure S4 and Table S1.

We examined the effects of loss of Fbxl7 on Ft and Ds localization. This revealed a small decrease in Ft (and possibly Ds) levels at junctions, but no significant decrease in polarity (Figures 7A–7D). We also examined the effect of Fbxl7 on Ft and Ds junctional stability using FRAP. Loss of Fbxl7 causes a decrease in stable Ds-EGFP and (and possibly Ft-EGFP) in puncta (Figures 7E, 7F, S4A, and S4B; Table S1). Conversely, overexpression of Fbxl7 increases stable Ds-EGFP in puncta (Figures 7G and S4C; Table S1). These data argue against Fbxl7 acting in feedback by stabilizing Ft and destabilizing Ds. However, feedback circuits can be subject to adaptation upon prolonged loss or gain of a component (Hoeller et al., 2014). We therefore examined the effects of temporally inducing Fbxl7 expression on Ds-EGFP stability, but again detected only an increase in stability (Figures 7H and S4D; Table S1).

These results support Fbxl7 playing a role in stabilizing both Ft and Ds at cell junctions, most likely via its binding to Ft. The failure to observe a reduction in cell polarity upon loss of Fbxl7, and the failure of Fbxl7 overexpression to destabilize Ds, both argue against a role in sorting of Ft-Ds heterodimers.

## Discussion

In this work we compare the mechanisms used to generate planar polarity by the core and Ft-Ds pathways, and assess the relative contributions of upstream global signals and feedback amplification.

In the core pathway, there is good evidence for feedback amplification (e.g., Strutt and Strutt, 2002; Strutt and Strutt, 2007; Strutt et al., 2011; Vladar et al., 2012; Cho et al., 2015; Aw et al., 2016; Warrington et al., 2017; Strutt et al., 2019). The core pathway proteins are found in clusters of highly aligned stable complexes, suggestive of active local sorting (Figure 1A″) (Strutt et al., 2011). Moreover, they can become asymmetrically localized to opposite cell edges to generate locally coordinated cell polarity in the apparent absence of global cues, implying the existence of non-local feedback acting at the cell scale (Figure 1A‴) (Strutt and Strutt, 2002; Strutt and Strutt, 2007; Vladar et al., 2012; Aw et al., 2016, and this work).

The presence of punctate Ft and Ds in developing wings (Ma et al., 2003; Hale et al., 2015) led us to seek evidence for local feedback interactions that might sort Ft-Ds heterodimers. We found that, as in the core pathway, punctate regions are sites of increased alignment of Ft-Ds heterodimers of the same orientation. Notably, some sorting persists in the absence of the Fj and Ds gradients that act as global cues, even though under these conditions cell-level polarity is lost. However, even in the presence of the gradients, the level of sorting in puncta is lower than seen for the core pathway, and FRAP analysis shows that heterodimers in the “wrong” orientation show similar stability to those in the “right” orientation.

From these results we make two proposals regarding mechanisms of planar polarization by the Ft-Ds pathway in the pupal wing: (1) upstream global cues in the form of graded expression of Fj and Ds are required for cellular planar polarization and there is an absence of non-local (cell-scale) feedbacks able to drive spontaneous cell polarization; (2) weak local feedback interactions partially sort Ft-Ds heterodimers within puncta and most likely amplify cellular polarity. The presence of a significant stable fraction of Ds on the wrong side of puncta may favor such local feedback interactions being “positive” and stabilizing Ft-Ds heterodimers of the same orientation, although we cannot rule out “negative” interactions destabilizing heterodimers of opposite orientation.

A recent theoretical publication suggested that if Ft stability on junctions is dependent on local Ds concentration on the apposing junction and vice versa, then this will generate a non-linearity in Ft-Ds heterodimer stability that could act as a sorting mechanism (Singh et al., 2021). We speculate that such cooperative effects of local Ft and Ds concentration might occur if clustering of Ft-Ds occurs via both *trans*-heterodimerization between cells and *cis*-homodimerization within cells. A comparable combination of *cis*- and *trans*-interactions allows E-cadherin to form stable domains at cell-cell contacts through a phase transition mechanism leading to a lattice-like arrangement (Zhang et al., 2009; Wu et al., 2010; Harrison et al., 2011).

Several lines of evidence support such a mechanism. First, both *Drosophila* and mammalian Ft-Ds homologs show strong heterophilic but undetectable homophilic *trans*-interactions (Matakatsu and Blair, 2004; Ishiuchi et al., 2009; Loza et al., 2017). Second, the Ft intracellular domain shows *cis*-interactions (Sopko et al., 2009). Third, in cultured cells, mammalian Fat4 and Ds1 proteins exhibit cooperativity of Ft-Ds binding and clustering of heterodimers of the same orientation (Loza et al., 2017), consistent with incorporation of heterodimers of the same orientation into puncta being favored over that of heterodimers in the opposite orientation.

Overall, our data reveal two distinct mechanisms for planar polarity establishment. The first, employed by the core pathway, involves cellular asymmetry being generated via feedback-mediated self-organization, with global cues being required only to orient the direction of polarization. The second, exemplified by the Ft-Ds pathway, requires initial establishment of cellular asymmetries in protein distribution through the presence of graded expression of pathway components or their regulators (Ds and Fj in this case), potentially followed by amplification of these asymmetries.

It may be biologically advantageous during development to have available these two different circuit logics of strong feedback/weak global cues versus strong global cues/weak feedback. The former logic most likely requires more complex cellular machinery to implement positive and negative feedbacks at different scales (Harrison et al., 2020), but can provide strong cellular polarity even when oriented by weak/transient upstream cues (e.g., Amonlirdviman et al., 2005; Burak and Shraiman, 2009; Schamberg et al., 2010). Conversely, strong global cues/weak feedback is likely to be simpler to implement—the Ft-Ds system may generate planar polarity through the activities of just Ft-Ds plus the regulatory kinase Fj (e.g., Jolly et al., 2014; Hale et al., 2015; Wortman et al., 2017). Furthermore, with weak or no amplification, the final polarity is likely to reflect the slope of the global gradient cues (see, e.g., Fisher and Strutt, 2019). This provides a way for cells to read and respond to not just gradient direction but also gradient steepness (Lawrence et al., 2008). Degree of polarity in the Ft-Ds pathway is known to regulate localization of the atypical myosin Dachs and through this control rates of cell division (Rogulja et al., 2008; Willecke et al., 2008), cell shape, and cell rearrangements (Mao et al., 2011; Bosveld et al., 2012). Hence, weak/no amplification may be a deliberate strategy to enable gradient control of morphogenesis.

### Limitations of the study

The aim of this work has been to examine the mechanisms of polarization used by the core and Ft-Ds pathways under as similar conditions as possible. To this end, we have compared their behaviors at the same stage of development in the same region of the wing, using measures of polarity that we have tested to be insensitive to differences in cell shape/size and image parameters (Tan et al., 2021). Nevertheless, we cannot rule out that some differences may arise due to the characteristics of reagents (antibodies and tagged proteins) used to detect different protein distributions. We also cannot exclude that the pathways show different behaviors at different stages of development, although both do appear important for pupal wing morphogenesis around the stage that we study (Merkel et al., 2014).

A further caveat is that the global cues that promote core pathway proximodistal polarity are poorly understood (see, e.g., Aw and Devenport, 2017; Ewen-Campen et al., 2020; Yu et al., 2020). The swirling polarity patterns seen upon late pathway activation cause us to speculate that these cues are only present earlier in pupal wing development (Strutt and Strutt, 2002, 2007). However, we cannot exclude that weak or residual global cues contribute to local polarity coordination in our *de novo* polarization experiments.

Finally, the weak sorting in puncta observed in the absence of global cues for the Ft-Ds pathway cannot be taken as proof that there is cellular amplification of polarity in this pathway. In fact, Ft-Ds polarity might be entirely dependent on the steepness of the Fj and Ds gradients, with no contribution of feedback amplification. Further dissection of the feedback mechanisms will be required to fully understand their roles.

## STAR★Methods

### Key resources table

**Table:** 

REAGENT or RESOURCE	SOURCE	IDENTIFIER
**Antibodies**		

Mouse monoclonal anti-Fmi (#74)	Developmental Studies Hybridoma Bank (Usui et al., 1999)	RRID:AB_2619583
Rabbit anti-Ds affinity purified	(Strutt and Strutt, 2002)	N/A
Rat anti-Ft serum	(Brittle et al., 2010)	N/A
Rabbit anti-Fj affinity purified	(Hale and Strutt, 2015)	N/A

**Experimental models: Organisms/strains**		

*fz-EGFP*	(Strutt et al., 2016)	FLYB: FBti0206968
*ft-EGFP*	(Hale et al., 2015)	N/A
*ds-EGFP*	(Brittle et al., 2012)	FLYB: FBti0202074
*ds-mApple*	This study	N/A
*P[acman]-EGFP-stbm attP40 (2L) 25C6*	(Strutt et al., 2019)	FLYB: FBtp0133033
*P[acman]-mApple-stbm attP40 (2L) 25C6*	(Strutt et al., 2019)	FLYB: FBtp0137434
*P[acman]-Fbxl7-mEGFP VK02 (2L) 28E7*	This study	N/A
*P[acman]-mCherry-Fbxl7 VK02 (2L) 28E7*	This study	N/A
*P[w+, Actin5C-FRT-PolyA-FRT-fz-EYFP*^*4.1*^*]*	(Strutt, 2001)	FLYB: FBtp0017633
*P[w+, arm-ds]*	This study	N/A
*P[w+, arm-FRT-PolyA-FRT-ds]*	This study	N/A
*P[w+, Actin5C-fj] attP2*	This study	N/A
*y w P[w+, UbxFLP]1*	Bloomington Drosophila Stock Center (Emery et al., 2005)	BDSC:42718; FLYB:FBti0150334; RRID:BDSC_42718
*y w; P[w+, UbxFLP]3*	Bloomington Drosophila Stock Center (Emery et al., 2005)	BDSC: 42718; FLYB: FBti0150356; RRID:BDSC_42719
*P[ry+, hsFLP]*	Bloomington Drosophila Stock Center (Golic and Lindquist, 1989)	BDSC:6; FLYB:FBti0002044; RRID:BDSC_6
*FRT40*	Bloomington Drosophila Stock Center (Xu and Rubin, 1993)	FLYB: FBti0002071
*FRT80*	Bloomington Drosophila Stock Center (Xu and Rubin, 1993)	FLYB: FBti0002073
*FRT82*	Bloomington Drosophila Stock Center (Xu and Rubin, 1993)	FLYB:FBti0002074
*P[w+, arm-lacZ] FRT40*	Bloomington Drosophila Stock Center	BDSC:7371; RRID:BDSC_7371
*P[w+, arm-lacZ] FRT80*	Bloomington Drosophila Stock Center	BDSC:6341; RRID:BDSC_6341
*FRT82 P[w+, arm-lacZ]*	Bloomington Drosophila Stock Center	BDSC:7369; RRID:BDSC_7369
*P[w+, ptc-GAL4]*	Bloomington Drosophila Stock Center (Hinz et al., 1994)	BDSC:2017; FLYB:FBti0002124; RRID:BDSC_2017
*P[w+, Act-GAL4]*	Bloomington Drosophila Stock Center	BDSC:4414; FLYB:FBti0012293; RRID:BDSC_4414
*P[w+, tub-GAL80*^*ts*^*]*	Bloomington Drosophila Stock Center	BDSC:7019; FLYB:FBti0027796; RRID:BDSC_7019
*fz*^*P21*^	(Jones et al., 1996)	BDSC:41787; FLYB:FBal0004937; RRID:BDSC_41787
*stbm*^*6*^	(Wolff and Rubin, 1998)	BDSC:6918; FLYB:FBal006242; RRID:BDSC_6918
*fj*^*d1*^	(Villano and Katz, 1995)	BDSC:6373; FLYB:FBal0049500; RRID:BDSC_6373
*ds*^*UA071*^	(Adler et al., 1998)	BDSC:41784; FLYB:FBal0089339; RRID:BDSC_41784
*ds*^*38k*^	(Clark et al., 1995)	BDSC:288; FLYB:FBal0028156; RRID:BDSC_288
*fbxl7*^*Q201X*^	(Bosch et al., 2014)	FLYB:FBal0298383
*P[w+, UAS-FLAG-fbxl7]*	(Bosch et al., 2014)	FLYB:FBal0298386

**Software and algorithms**		

ImageJ		https://imagej.nih.gov/ij/
MATLAB		https://uk.mathworks.com/products/matlab.html
Puncta selection script (MATLAB)		(Strutt et al., 2016)
GraphPad Prism v8		https://www.graphpad.com
G^∗^Power3.1		https://www.psychologie.hhu.de/arbeitsgruppen/allgemeine-psychologie-und-arbeitspsychologie/gpower
QuantifyPolarity		(Tan et al., 2021)

### Resource availability

#### Lead contact

Further information and requests for resources and reagents should be directed to and will be fulfilled by the lead contact, David Strutt (d.strutt@sheffield.ac.uk).

#### Materials availability

Fly strains generated in this study will be made available on request to the lead contact.

### Experimental model and subject details

#### *Drosophila* genetics

*Drosophila melanogaster* flies were grown on standard cornmeal/agar/molasses media and all experiments performed at 25°C except for GAL4/UAS overexpression experiments at 18°C/29°C as described in figure legends. Fly strains are described in the Key resources table.

Genotypes for each figure

Figure 2

(B, F, I, L) *y w P[w+, UbxFLP]1; P[w+, arm-lacZ] FRT40/ds-EGFP FRT40*

(C, G, J, M) *y w P[w+, UbxFLP]1; P[w+, arm-lacZ] FRT40/ft-EGFP FRT40*

(D, E, H, K) *y w P[w+, UbxFLP]1; P[w+, arm-lacZ] FRT80/fz-EGFP FRT80*

Figure 3

(A) *y w P[w+, UbxFLP]1; ft-EGFP FRT40/ds-mApple FRT40*

(B, C, D, E) *y w P[w+, UbxFLP]1; P[w+, arm-lacZ] FRT40/ds-EGFP FRT40*

(F, G, H, I) *y w P[w+, UbxFLP]1; P[w+, arm-lacZ] FRT80/fz-EGFP FRT80*

Figure 4

(C, E) *y w P[w+, UbxFLP]1; ds-mApple FRT40/ds-EGFP FRT40*

(D, F) *y w P[w+, UbxFLP]1*; *P[acman]-EGFP-stbm FRT40 stbm*^*6*^*/P[acman]-mApple-stbm FRT40 stbm*^*6*^

Figure 5

(A-D) *w P[ry+, hsFLP]; P[w+, Actin5C-FRT-PolyA-FRT-fz-EYFP*^*4.1*^*]/+; fz*^*P21*^

(E-H) *w P[ry+, hsFLP]; ds*^*UA071*^ *P[w+, arm-FRT-PolyA-FRT-ds]/ds*^*UA071*^

(I-L) *w P[ry+, hsFLP]; ds*^*UA071*^ *P[w+, arm-FRT-PolyA-FRT-ds] fj*^*d1*^*/ds*^*UA071*^ *fj*^*d1*^*; P[w+, Actin5C-fj]/+*

Figure 6

(A, C, E-G) *y w P[w+, UbxFLP]1; P[acman]-mCherry-Fbxl7 FRT40/P[acman]-Fbxl7-mEGFP FRT40*

(B, D-F, H) *w; ds*^*38K*^ *P[acman]-mCherry-Fbxl7 FRT40 fj*^*d1*^*/ds*^*UA071*^ *P[acman]-Fbxl7-mEGFP FRT40 fj*^*d1*^*; P[w+, Actin5C-fj]/P[w+, arm-ds], P[w+, UbxFLP]3*

Figure 7

(A-D) *y w P[w+, UbxFLP]1; FRT82 fbxl7*^*Q201*^*/FRT82 P[w+, arm-lacZ]*

(E) *ds-EGFP/ds-EGFP* (controls), *ds-EGFP/ds-EGFP; FRT82 fbxl7*^*Q201X*^*/FRT82 fbxl7*^*Q201X*^ (experimental)

(F) *ft-EGFP/ft-EGFP* (controls), *ft-EGFP/ft-EGFP; FRT82 fbxl7*^*Q201X*^*/FRT82 fbxl7*^*Q201X*^ (experimental)

(G) *w; ds-EGFP P[w+, ptc-GAL4]/P[w+, UAS-FLAG-fbxl7]*

(H) *w; ds-EGFP/P[w+, Act-GAL4], P[w+, tub-GAL80*^*ts*^*]; P[w+, UAS-FLAG-fbxl7] FRT82 fbxl7*^*Q201X*^*/FRT82 fbxl7*^*Q201X*^ (induction experiments), *w; ds-EGFP/+; P[w+, UAS-FLAG-fbxl7] FRT82 fbxl7*^*Q201X*^*/FRT82 fbxl7*^*Q201X*^ (no induction controls)

Figure S1

(A, D, H) *y w P[w+, UbxFLP]1; P[w+, arm-lacZ] FRT80/fz-EGFP FRT80*

(B, E, G) *y w P[w+, UbxFLP]1; P[w+, arm-lacZ] FRT40/ds-EGFP FRT40*

(C, F) *y w P[w+, UbxFLP]1; P[w+, arm-lacZ] FRT40/ft-EGFP FRT40*

Figure S2

(A) *y w P[w+, UbxFLP]1; ft-EGFP FRT40/ds-mApple FRT40*

(B) *y w P[w+, UbxFLP]1; ds-mApple FRT40/ds-EGFP FRT40*

Figure S3

(B) *w[1118]*

(C) *w P[ry+, hsFLP]; ds*^*UA071*^ *fj*^*d1*^ *P[w+, arm-FRT-PolyA-FRT-ds]/ds*^*UA071*^ *fj*^*d1*^*; Act5C-fj/+* (with no heat-shock so *arm-d*s not expressed)

(D-G) *w P[ry+, hsFLP]; P[w+, Actin5C-FRT-PolyA-FRT-fz-EYFP*^*4.1*^*]/+; fz*^*P21*^

(H) *w[1118]*

(I) *w; ds*^*UA071*^ *P[w+, arm-ds]/ds*^*UA071*^

(J-M) *w P[ry+, hsFLP]; ds*^*UA071*^ *P[w+, arm-FRT-PolyA-FRT-ds]/ds*^*UA071*^

(N-Q) *w P[ry+, hsFLP]; ds*^*UA071*^ *P[w+, arm-FRT-PolyA-FRT-ds] fj*^*d1*^*/ds*^*UA071*^ *fj*^*d1*^*; P[w+, Actin5C-fj]/+*

Figure S4

(A) *ds-EGFP/ds-EGFP* (controls), *ds-EGFP/ds-EGFP; FRT82 fbxl7*^*Q201X*^*/FRT82 fbxl7*^*Q201X*^ (experimental)

(B) *ft-EGFP/ft-EGFP* (controls), *ft-EGFP/ft-EGFP; FRT82 fbxl7*^*Q201X*^*/FRT82 fbxl7*^*Q201X*^ (experimental)

(C) *w; ds-EGFP P[w+, ptc-GAL4]/P[w+, UAS-FLAG-fbxl7]*

(D) *w; ds-EGFP/P[w+, Act-GAL4], P[w+, tub-GAL80*^*ts*^*]; P[w+, UAS-FLAG-fbxl7] FRT82 fbxl7*^*Q201X*^*/FRT82 fbxl7*^*Q201X*^ (induction experiments), *w; ds-EGFP/+; P[w+, UAS-FLAG-fbxl7] FRT82 fbxl7*^*Q201X*^*/FRT82 fbxl7*^*Q201X*^ (no induction controls)

### Method details

#### Transgenic strain generation

Novel transgenic fly lines were generated by injection of constructs by BestGene or Genetivision. Uniform *ds* expression was achieved by cloning the *ds* CDS downstream of the *arm* promoter and randomly integrating into the genome by P-element transgenesis (*P[w+, arm-ds]*), and for inducible expression a removable stop cassette was included between the promoter and the CDS (*P[w+, arm-FRT-PolyA-FRT-ds]*). Uniform *fj* expression was achieved by cloning the *fj* CDS into the vector *attB-Actin5C>stop>polyA* vector (Strutt et al., 2013), transforming into *attP2* and removing the stop cassette via expression of FLP recombinase. To generate a functional mApple fusion to the C-terminus of the *ds* CDS, we first inserted an attP site into the *ds* locus that deletes from 50 bp upstream of the ATG to an intronic position 1.9 kb downstream, using the targeting vector pTV[Cherry] (Baena-Lopez et al., 2013). A *ds* cDNA fused to the mApple CDS with 50 bp of 5′ UTR upstream of the *ds* ATG and the complete *ds* 3′UTR was then inserted into the attP site in a modified version of the vector pGE-attB-GMR (Huang et al., 2009). Fusion of mEGFP and mCherry coding sequences to the Fbxl7 CDS was achieved using standard recombineering methods (Venken et al., 2008) starting from P[acman]-CmR-CH322-07N06, and introduced into attP site VK02 on 2L. Maps and further details are available upon request.

#### Immunolabelling

Dissection and staining of pupal wings and wing discs was performed as previously reported (Strutt, 2001; Brittle et al., 2012). Briefly, pupae were fixed in 4% paraformaldehyde in PBS and pupal wings dissected away, with a total fixation time of 30–40 min at room temperature. Wing discs were fixed in 4% PFA for 20 min on the larval cuticle and then dissected off after immunolabelling. Pupal wings or discs were blocked for 30–60 min in PBS containing 0.2% Triton X-100 (PTX) and 10% normal goat serum prior to antibody incubation. Primary antibodies were incubated overnight at 4°C, and secondary antibodies either for 2–4 h at room temperature or overnight at 4°C, in PTX with 10% normal goat serum, all washes were in PTX. After immunolabelling, pupal wings were mounted in 10% glycerol, 1xPBS, containing 2.5% DABCO (pH7.5) and wing discs were mounted in Moviol/DABCO. For resolution doubling imaging using the AiryScan, wing discs were mounted in Vectashield. Primary antibodies for immunolabelling were mouse monoclonal anti-Fmi (Flamingo #74, DSHB, Usui et al., 1999), rabbit anti-Ds affinity purified polyclonal (Strutt and Strutt, 2002), rat anti-Ft polyclonal (Brittle et al., 2010), rabbit anti-Fj affinity purified polyclonal (Hale et al., 2015).

#### Imaging of fixed samples

Pupal wings and wing discs were imaged on a Nikon A1R GaAsP confocal microscope using a 60× NA1.4 apochromatic lens. Z-slices separated by 150 nm were imaged at a pixel size of 80–100 nm, and the three brightest slices around apicolateral junctions were selected and averaged for each channel in ImageJ. To image Fj localisation in the whole of the region posterior to vein 4, Z slices were separated by 200 nm and imaged at a pixel size of 200 nm and stacks maximum projected in ImageJ. Resolution doubling imaging was carried out using a Zeiss LSM 880 AiryScan with a 63× lens NA 1.4 apochromatic lens. In the pupal wing all images were taken posterior to vein 4 with the images oriented with vein 4 at the top of the image. In the wing disc images were taken in the dorsal half of the disc close to the anteroposterior compartment boundary.

#### Live imaging and FRAP

For live imaging, pupae were prepared and wings imaged at 28 h APF and third instar wing discs dissected, mounted and imaged as previously described (Strutt et al., 2011; Hale et al., 2015). For FRAP, regions of interest (ROIs) of 2 μm in size were selected for puncta.

#### Induction experiments

*hsFLP* was used to excise the FRT-STOP-FRT cassette to allow expression of Ds and Fz-EYFP (*P[w+, arm-FRT-PolyA-FRT-ds]*, *P[w+, Actin5C-FRT-PolyA-FRT-fz-EYFP]*). Pupae were heat-shocked for 2 h at 38°C at the indicated times. On the basis of when trichomes subsequently emerge, we observe that development is halted for the period of the heat shock. The time of induction of protein expression was varied but the tissue fixed at the same developmental time of 28 h APF.

Temporally controlled induction of Fbxl7 expression was achieved using the GAL4/GAL80^ts^ system (McGuire et al., 2003). Larvae were incubated at 18°C (no expression condition) then shifted to 29°C for the indicated period of time to induce expression.

### Quantification and statistical analysis

#### Measuring intensity on clone boundaries and in puncta/non-puncta

To measure fluorescent protein intensity on whole junctions on clone boundaries junctions were selected manually in ImageJ (5 pixel width line) and labeled with their orientation (proximal/distal/lateral or anterior/posterior/lateral) dependent upon the angle of the line (in 90° bins) relative to vein 4 which was oriented as 0°. Clone regions lacking EGFP were measured and used to subtract background signal. Mean intensity for the oriented junctions were calculated per wing image.

To measure intensity in puncta and non-puncta regions, samples were co-immunolabelled with Fmi or Ds and the images analysed to select puncta by total area using a Matlab script (Strutt et al., 2016). Selecting 4% of the image as puncta picked out all the brightest puncta. Puncta on clone boundaries were selected manually and labelled with the orientation of the junction. Non-puncta regions were manually selected as regions lying between puncta. Regions were measured using a square box of size 0.32 μm^2^ (pupal wings) or 0.16 μm^2^ (wing disc) that covered the width of the junctions. Mean intensity per wing was then calculated.

Mean intensities were compared using a one-way ANOVA. Tukey-Kramer’s multiple comparison test was used to compare between all categories of junction within an experiment and Sidak’s multiple comparison test was used to compare genotypes pairwise. Where a post hoc test was used this is described in the figure legends, and multiplicity adjusted p values calculated in GraphPad Prism are indicated on the graphs. Based on the mean junctional asymmetry of Ds-EGFP in a pilot experiment, we aimed for a sample size of at least 6 wings per timepoint. This would allow detection of differences of 20% in the means, in a pairwise comparison, with a power of 0.8 and α 0.05 (using G^∗^Power3.1).

To test the function of endogenously tagged Ft-EGFP and Ds-EGFP, twin-clones of EGFP tagged and untagged tissue were generated and the distribution of tagged and untagged protein examined. Proteins were immunolabelled for Ft or Ds and the puncta, membrane non-puncta and intracellular intensity of signal of tagged and untagged protein measured. There was no significant difference in localisation of tagged and untagged Ft. A 10% difference in the intensity of EGFP tagged Ds compared to untagged Ds was found. Fz-EGFP was previously characterised (Strutt et al., 2016). Polarity measurements of tagged and untagged proteins were also compared with only Ds-EGFP differing significantly showing a small increase (paired t test p = 0.03) in neighbour vector average polarity (untagged = 0.07010 and tagged = 0.08031). Therefore, tagging Ft and Ds with EGFP does not greatly alter the ability of the proteins to localise and polarise.

#### Puncta heterogeneity measurements

For the puncta heterogeneity experiments, puncta were selected by total area as above using Ds antibody staining and a Matlab script. Levels of both mEGFP and mCherry were measured in puncta on twin-clone boundaries.

For each wing, the levels of mEGFP and mCherry intensities for puncta i on clone boundaries were normalised against the average fluorescence intensities of all the puncta:

$$I_{\text{n}\_{\text{mEGFP}}_{i}}=\frac{I_{{\text{mEGFP}}_{i}}}{\left[\frac{1}{m}\sum_{j=1}^{m}I_{{\text{mEGFP}}_{i}}\right]}\;,$$

$$I_{\text{n}\_{\text{mCherry}}_{i}}=\frac{I_{{\text{mCherry}}_{i}}}{\left[\frac{1}{m}\sum_{j=1}^{m}I_{{\text{mCherry}}_{i}}\right]},$$

where $I_{{\text{mEGFP}}_{i}}$ and $I_{{\text{mCherry}}_{i}}$ are the fluorescence intensities for mEGFP and mCherry of puncta i, respectively, m is the total number of puncta, $I_{\text{n}\_{\text{X}}_{i}}$ denotes normalised fluorescence intensity of compound X and puncta i, with X= mEGFP, mCherry. Similar normalisation was performed for control puncta in heterozygous tissue. In cases where intensities of only anterior and posterior puncta were measured, normalisation was carried out only against the measured puncta, excluding the lateral puncta.

To determine the level of sorting in puncta on clone boundaries, we first computed the ratio of normalised mEGFP to mCherry intensities for each puncta on twin-clone boundaries, denoted by $\;r_{{\text{CB}}_{i}}$ as follows:

$$\;r_{{\text{CB}}_{i}}=\frac{I_{\text{n}\_{\text{mEGFP}}_{i}}}{I_{\text{n}\_{\text{mCherry}}_{i}}}\;.$$

The average ratio for control puncta in heterozygous tissue, $\;r_{\text{het}}$, was determined by fitting a line onto mEGFP vs mCherry data. Then, the gradient of the fitted line is the average ratio of the control puncta from heterozygous tissue and it is determined as follows:

$$x^{2}=\;\sum_{j=1}^{m}{\tilde{I}}_{\text{n}\_{\text{mEGFP}}_{i}}^{2},\;y^{2}=\sum_{j=1}^{m}{\tilde{I}}_{\text{n}\_{\text{mCherry}}_{i}}^{2},\;xy=\sum_{j=1}^{m}{\tilde{I}}_{\text{n}\_{\text{mEGFP}}_{i}}\cdot {\tilde{I}}_{\text{n}\_{\text{mCherry}}_{i}},$$

$$r_{\text{het}}=\frac{1}{2xy}\left[\sqrt{4{\left(xy\right)}^{2}+{\left(x^{2}-y^{2}\right)}^{2}}+\left(y^{2}-x^{2}\right)\right],$$

where tilde denotes the normalised intensities in heterozygous tissue.

The difference $D_{i}$ between the ratio on clone boundaries $r_{{\text{CB}}_{i}}$ and average ratio of the control puncta is calculated as follows:

$$D_{i}=\left\{ \begin{array}{ll} |\frac{1}{r_{\text{het}}}-r_{{\text{CB}}_{i}}|, & \text{if}\;r_{{\text{CB}}_{i}}<1\\ \left|r_{\text{het}}-r_{{\text{CB}}_{i}}\right|, & \text{otherwise} \end{array}\right..$$

Finally, the average difference $\bar{D}$ for each wing which is termed as ‘puncta heterogeneity’ value, is determined as follows:

$$\;\bar{D}=\frac{1}{m}\sum_{j=1}^{m}D_{i}$$

Puncta heterogeneity value $\bar{D}$ approaches 1 if proteins within the puncta are well-sorted/separated and approaches 0 if proteins within the puncta are well mixed. The puncta sorting values from multiple wings and different pupae (n, number of wings) are compared using an unpaired t test.

#### Polarity measurements

To measure polarity, wing images were aligned along the proximodistal wing axis based on wing vein orientation, and membrane masks were generated using Packing Analyzer (Aigouy et al., 2010).

All polarity measurements were obtained using QuantifyPolarity Graphical User Interface (Tan et al., 2021). Polarity magnitude and angle of a single cell are computed using Principle Component Analysis (PCA) based method, which computes the largest variance of weighted intensities from the centroid of the cell. Within a group of cells, the polarity measurements can be combined in specific ways to reveal strength of polarity as well as polarity coordination between cells. The most direct way is to compute the average of polarity magnitudes of each cell without considering average polarity angle, which is termed ‘average polarity magnitude’. In addition, ‘average neighbour vector polarity’ is used to assess the strength of coordination of polarity between a cell and its immediate neighbours by taking the vector average of the polarity vector for this group of cells. We use this measure to assess local coordination of polarity as it measures the coordination over a small region (usually 7 cells in a hexagonally packed tissue) and thus will report a swirling polarity pattern as being locally coordinated. We do not use the alternative measure of ‘vector average polarity magnitude’ where vectors are averaged over the entire image region as this reports swirling polarity patterns as poorly coordinated. For more explanation, see summary in Figure S6 of Tan et al. (2021).

To visualise the cell polarity angle, we plotted circular weighted histograms as described (Tan et al., 2021). Briefly, polarity magnitude and angle are obtained on a cell-by-cell basis from the QuantifyPolarity Graphical User Interface. Data from multiple wings are combined and grouped into 20 bins, with each bin representing a range of polarity angles. Histograms are weighted by the average polarity magnitude of the given bin. Hence, the radial length of each bin indicates the weighted frequency of occurrences, while the orientation of each bin represents the angle of average polarity. All computed polarity angles are plotted in a range between 180° and 360°, with 180°/360° corresponding to the horizontal axis of the image.

For the polarity analysis following induction of gene expression, a cropped 300 × 300 pixel (31.5 × 31.5 μm) region was selected in the centre of the image at least 5 cells away from vein 4. Based on the mean cell polarity measurements of a pilot experiment, we aimed for a sample size of at least 6 wings per timepoint. This would allow detection of differences of 20% in the means, in a pairwise comparison, with a power of 0.8 and α 0.05 (using G^∗^Power3.1). This was achieved for all but one timepoint where we lacked 1 sample.

#### FRAP analysis

After imaging of a FRAP experiment, ROIs were manually reselected in ImageJ and quantitated. Control unbleached regions were also quantitated to control for acquisition bleaching. Data were corrected for acquisition bleaching and normalised against an average of the prebFleached values and the first postbleach value. Data from ROIs in the same wing were averaged. Prism (v8 GraphPad) was used to fit a one-phase exponential association curve for each wing. Data from several wings were then used to fit a final exponential association curve, and an extra sum-of-squares F test was performed to compare curve plateaux (Ymax) between puncta and non-puncta or between genotypes. To determine the stable amount of EGFP in the ROIs, the mean intensity of the ROIs from the three pre-bleach images was measured in ImageJ, and averaged per wing. The intensity was then corrected for distance from the coverslip as previously described (Strutt et al., 2016), and this value was then multiplied by the stable fraction (1-y[max]) for each wing. The stable amounts were then averaged across wings. See Warrington et al. (2022) for further details.

## Data Availability

• Microscopy data reported in this paper will be shared upon request by the lead contact. • This paper does not report original code. • Any additional information required to reanalyse the data reported in this paper is available upon request from the lead contact.

## Supplemental information

Document S1. Figures S1–S4 and Table S1

Document S2. Article plus supplemental information
