# Supplementary material for: Distinct mechanisms of planar polarization by the core and Fat-Dachsous planar polarity pathways in the Drosophila wing
Source: Cell Rep. 2022 Sep 27;40(13):111419. doi: 10.1016/j.celrep.2022.111419 (PMC9631118; doi:10.1016/j.celrep.2022.111419)
Supplement: Document S1. Figures S1–S4 and Table S1 [file mmc1.pdf]

Cell Reports, Volume *40*

Supplemental information

Distinct mechanisms of planar polarization by the core and Fat-Dachsous planar polarity pathways in the *Drosophila* wing

Amy Brittle, Samantha J. Warrington, Helen Strutt, Elizabeth Manning, Su Ee Tan, and David Strutt

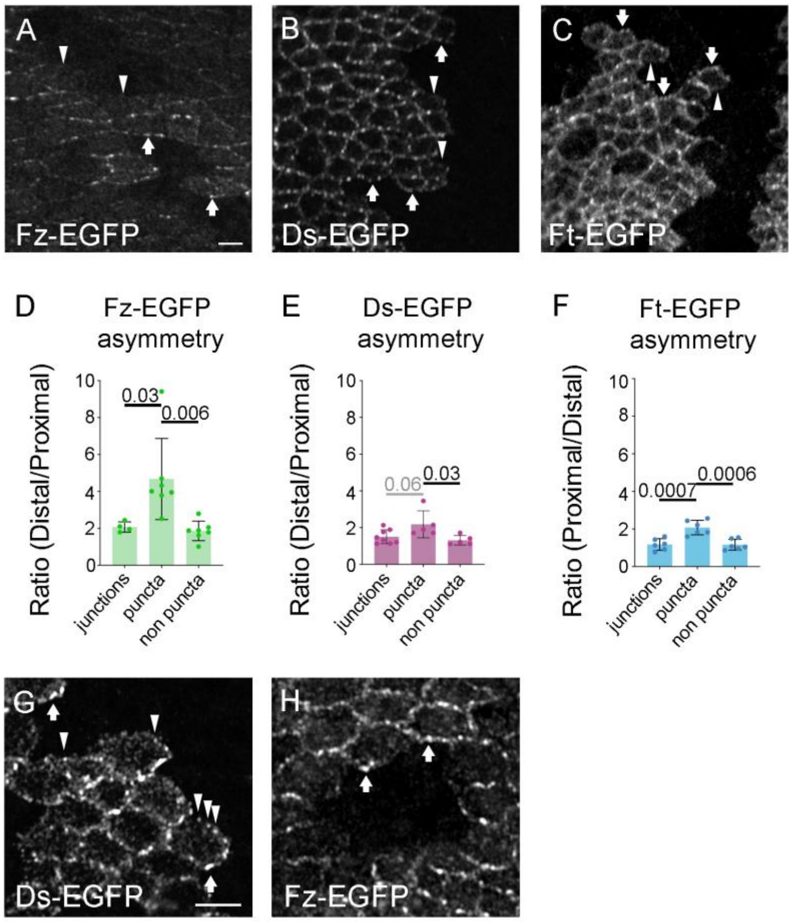

Supplemental Figure 1. Asymmetry of Fz, Ds and Ft in junctions, puncta and non-puncta in the wandering third instar larval wing disc. Related to Figure 2.

(A-C) Images of *fz-EGFP* (A), *ds-EGFP* (B) and *ft-EGFP* (C) twin-clones versus untagged protein, revealing asymmetric cellular localisations on clone boundaries. All images taken in the dorsal pouch region of the wing disc close to the hinge (dorsal/proximal up, ventral/distal down). Arrows point to high EGFP levels – Fz-EGFP (A) and Ds-EGFP (B) are high on distal junctions and Ft-EGFP (C) is higher on proximal cell junctions. Arrowheads point to low EGFP regions. Scale bar 5 µm.

(D-F) Asymmetry of EGFP in proximodistal junctions, puncta and non-puncta regions. Ratio of mean intensity of EGFP indicates asymmetry of protein. All means were compared with ANOVA analysis (Tukey's multiple comparisons test) and p-values indicated. Error bars are standard deviation.

(G,H) High resolution (AiryScan) images of Ds-EGFP and Fz-EGFP clone tissue in the wing disc. Images taken close to pouch-hinge boundary with distal at the bottom of images. Bright punctate distal Ds-EGFP (G) and Fz-EGFP (H) are seen (arrows) in accordance with the expected axis of polarisation at this developmental stage. Proximal Ds-EGFP in puncta can also be seen (arrowheads) but distinct proximal Fz-EGFP puncta are not evident.

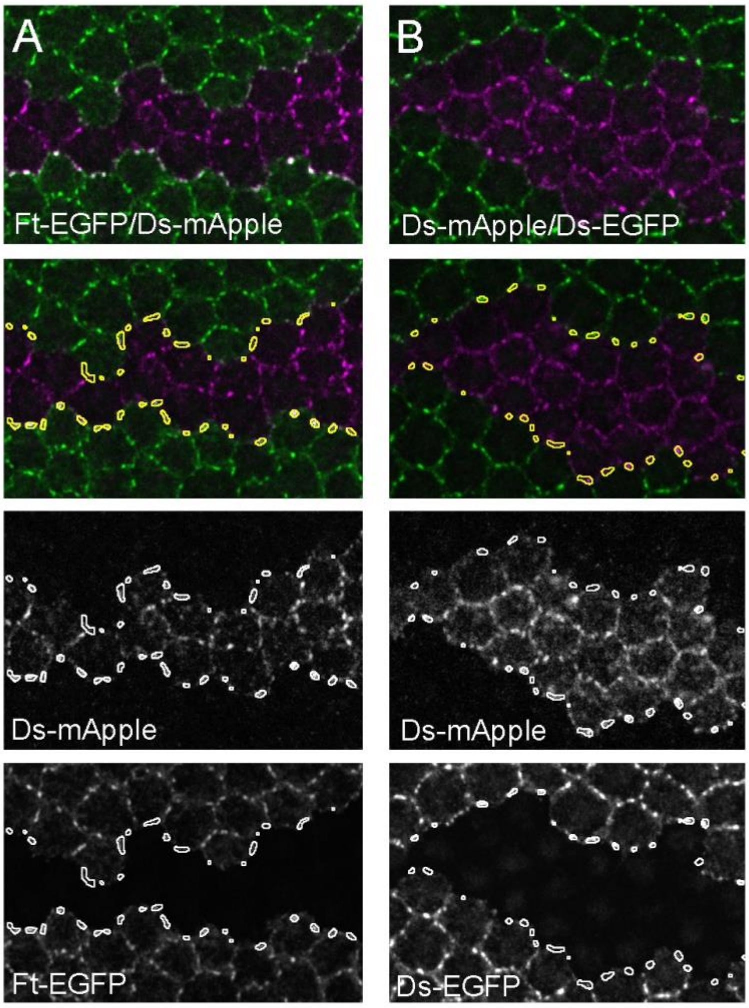

Supplemental Figure 2. EGFP and mApple tagged Ft and Ds proteins in apposed junctions are entirely localised within puncta regions selected using Ds immunolabelling. Related to Figure 4.

(A,B) Images of twin-clone tissue in 28 hr pupal wings showing *ft-EGFP/ds-mApple* (A) and *ds-EGFP/ds-mApple* (B). Puncta regions selected using thresholding (see STAR Methods) based on immunolabelling with Ds antibody are outlined in yellow or white on the images. Fluorescent signal of EGFP and mApple tagged Ft/Ds proteins in puncta on apposed junctions can be seen to be entirely contained within the selected regions.

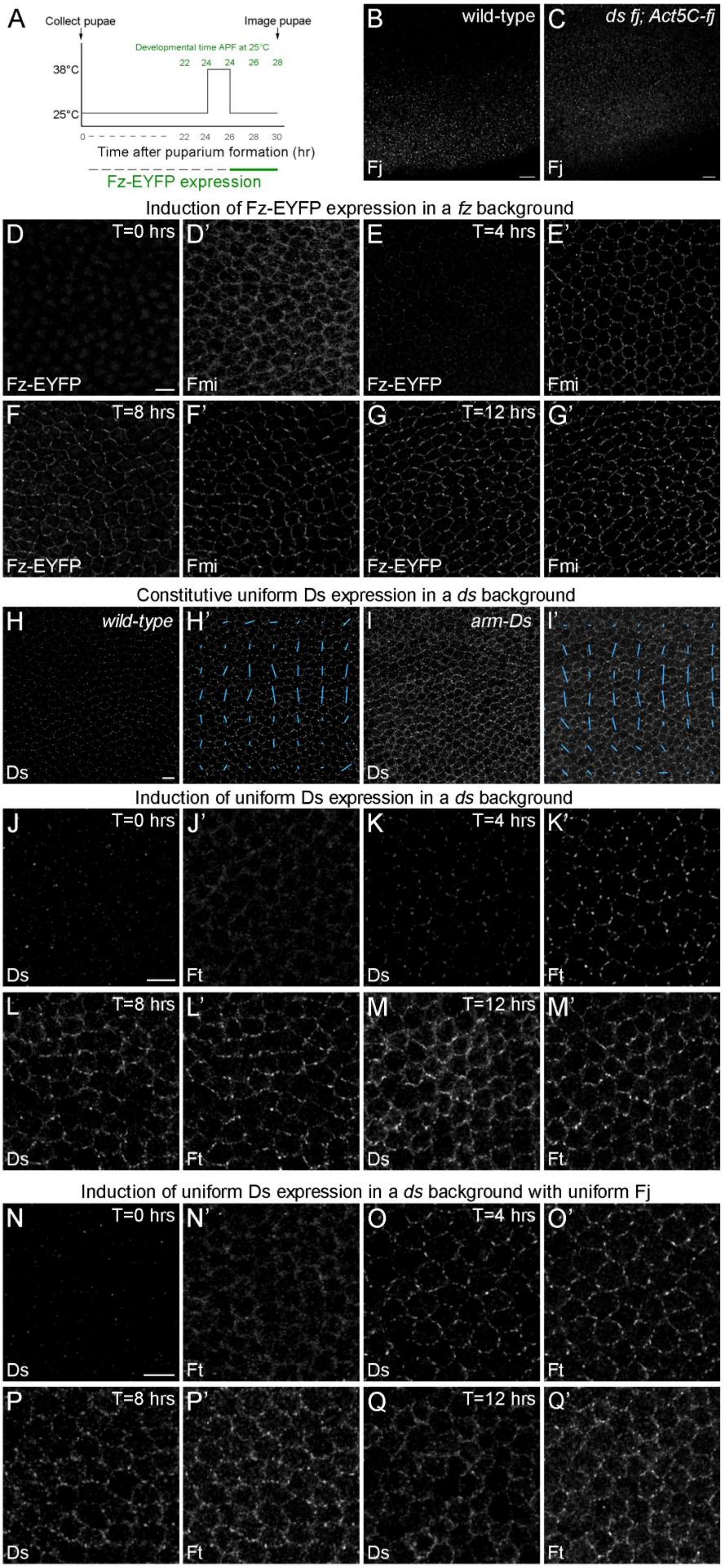
A
Collect pupae
Image pupae
Developmental time APF at 25°C
22 24 24 26 28
38°C
25°C
0 22 24 26 28 30
Time after puparium formation (hr)
Fz-EYFP expression
B wild-type
Fj
C ds fj; Act5C-fj
Fj
Induction of Fz-EYFP expression in a fz background
D T=0 hrs
Fz-EYFP
D' Fmi
E T=4 hrs
Fz-EYFP
E' Fmi
F T=8 hrs
Fz-EYFP
F' Fmi
G T=12 hrs
Fz-EYFP
G' Fmi
Constitutive uniform Ds expression in a ds background
H wild-type
Ds
H'
I arm-Ds
Ds
I'
Induction of uniform Ds expression in a ds background
J T=0 hrs
Ds
J' Ft
K T=4 hrs
Ds
K' Ft
L T=8 hrs
Ds
L' Ft
M T=12 hrs
Ds
M' Ft
Induction of uniform Ds expression in a ds background with uniform Fj
N T=0 hrs
Ds
N' Ft
O T=4 hrs
Ds
O' Ft
P T=8 hrs
Ds
P' Ft
Q T=12 hrs
Ds
Q' Ft

Supplemental Figure 3. The core pathway but not the Ft-Ds pathway *de novo* self-organises coordinated cellular polarity. Related to Figure 5.

(A) Timeline showing a heat-shock induction of Fz-EYFP expression relative to developmental time, with an example of induction of expression for 4 hr prior to dissection at 28 hr APF. Note, based on timing of trichome initiation, we observe that developmental time is halted during heat shock at 38°C.

(B, C) Images of 28 hr pupal wings, posterior to vein 4, immunolabelled for Fj. (B) Wild-type wing and (C) wing in which Fj is expressed uniformly under the *Act5C* promoter in a *fj* mutant background. Scale bar 10 µm.

(D-G) Images of 28 hr pupal wings during the time course of a Fz-EYFP induction experiment in a *fz* mutant background, demonstrating the turning on of Fz-EYFP expression (D-G) and Fmi localisation (D'-G'). Scale bar 5 µm.

(H, I) Images of 28 hr pupal wings below vein 4 immunolabelled for Ds and overlaid with coarse grain polarity of Ds (blue lines, H',I') in a wild-type background (H, H') and a wing constitutively expressing Ds under the *arm* promoter in a *ds* mutant background (B, B'). Ds expressed uniformly from the *arm* promoter still polarises on the anteroposterior axis indicating the sufficiency of the Fj gradient to act as a global cue. Scale bar 10 µm.

(J-M) Images of 28 hr pupal wings during a time course of a Ds induction experiment, demonstrating turning on of Ds expression in a *ds* background (J-M) and Ft localisation (J'-M'). Scale bar 5 µm.

(N-Q) Images from 28 hr pupal wings during a time course of a Ds induction experiment in a *ds* background also expressing uniform Fj, demonstrating the turning on of Ds expression (N-Q) and Ft localisation (N'-Q'). Scale bar 5 µm.

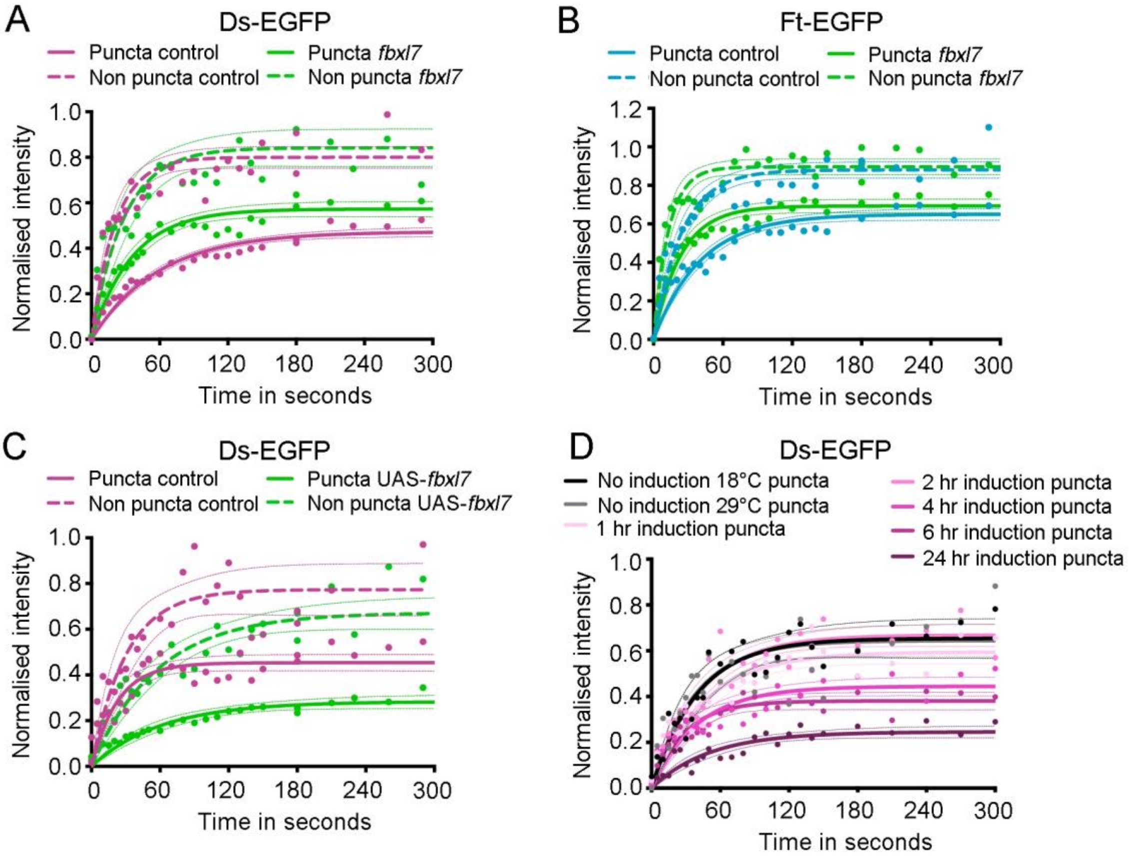

Supplemental Figure 4. Fbxl7 stabilises Ft-Ds in puncta. Related to Figure 7.

(A) FRAP analysis of Ds-EGFP in puncta and non-puncta regions of cell junctions in wing discs in control regions (purple) or *fbxl7* mutant tissue (green), showing recovery of EGFP intensity of bleached regions normalised to initial intensity. Faint lines show 95% confidence intervals for curves. Number of wings analysed n=6 for control regions, n=7 for *fbxl7* mutant tissue.

(B) FRAP analysis of Ft-EGFP in puncta and non-puncta regions of cell junctions in wing discs in control regions (teal) or *fbxl7* mutant tissue (green), showing recovery of EGFP intensity of bleached regions normalised to initial intensity. Faint lines show 95% confidence intervals for curves. Number of wings analysed n=5 for control regions, n=6 for *fbxl7* mutant tissue.

(C) FRAP analysis of Ds-EGFP in puncta and non-puncta regions of cell junctions in wing discs in control regions (purple) or *fbxl7* overexpression tissue (green) at 29°C, showing recovery of EGFP intensity of bleached regions normalised to initial intensity. Faint lines show 95% confidence intervals for curves. Number of wings analysed n=3 for control regions, n=5 for *fbxl7* overexpression tissue.

(D) FRAP analysis of Ds-EGFP in puncta regions of cell junctions in wing discs with *UAS-fbxl7* overexpression temporally induced in an *fbxl7* mutant background using *Act-GAL4,tub-GAL80*^{ts} by shifting animals from 18°C to 29°C for the time period indicated, showing recovery of EGFP intensity of bleached regions normalised to initial intensity. Control tissue without overexpression at 18°C is shown in black and 29°C in grey. Faint lines show 95% confidence intervals for curves.

Supplemental Table 1. FRAP data with 95% confidence intervals. Related to Figures 3 and 7.

	Half-life (in seconds) and 95% confidence intervals	y[max] and 95% confidence intervals	Stable amount (arbitrary fluorescence units) and 95% confidence intervals
**Figure 3B and C**			
Ds-EGFP anterior junctions	19.64 (15.62 to 26.46)	0.756 (0.718 to 0.793)	198.9 (24.74 to 373.1)
Ds-EGFP posterior junctions	28.99 (24.56 to 35.36)	0.601 (0.576 to 0.625)	442.7 (271.3 to 614.1)
**Figure 3D and E**			
Ds-EGFP anterior puncta	17.08 (12.61 to 26.46)	0.673 (0.631 to 0.715)	361.9 (49.70 to 674.0)
Ds-EGFP posterior puncta	24.92 (20.23 to 32.45)	0.633 (0.603 to 0.663)	693.5 (413.0 to 974.0)
**Figure 3F and G**			
Fz-EGFP proximal junctions	23.82 (18.22 to 34.40)	0.907 (0.816 to 0.963)	64.08 (-132.8 to 261.0)
Fz-EGFP distal junctions	31.58 (26.08 to 40.02)	0.286 (0.272 to 0.2993)	1826 (1609 to 2044)
**Figure 3H and I**			
Fz-EGFP proximal puncta	14.07 (9.894 to 24.33)	0.718 (0.666 to 0.770)	78.27 (17.79 to 138.8)
Fz-EGFP distal puncta	36.35 (29.21 to 48.12)	0.315 (0.295 to 0.334)	1712 (929.0 to 2495)
**Figure 7E**			
Ds-EGFP puncta	40.70 (35.95 to 46.89)	0.473 (0.452 to 0.494)	646.8 (479.4 to 814.2)
Ds-EGFP non puncta	14.43 (11.24 to 20.15)	0.800 (0.752 to 0.848)	88.00 (14.21 to 161.8)
Ds-EGFP *fbxl7* puncta	24.75 (20.46 to 31.32)	0.573 (0.540 to 0.606)	432.0 (22.12 to 236.9)
Ds-EGFP *fbxl7* non puncta	19.74 (14.11 to 32.84)	0.842 (0.759 to 0.925)	129.5 (22.12 to 236.9)
**Figure 7F**			
Ft-EGFP puncta	28.24 (24.35 to 33.60)	0.650 (0.620 to 0.680)	718 (372.2 to 1065)
Ft-EGFP non puncta	16.89 (13.82 to 21.72)	0.880 (0.838 to 0.923)	160.8 (-86.18 to 407.7)
Ft-EGFP *fbxl7* puncta	16.38 (13.40 to 21.06)	0.693 (0.658 to 0.728)	628.2 (381.5 to 874.9)
Ft-EGFP *fbxl7* non puncta	9.03 (7.11 to 12.37)	0.896 (0.856 to 0.937)	72.91 (-64.47 to 310.4)
**Figure 7G**			
Ds-EGFP puncta	15.94 (11.84 to 24.38)	0.454 (0.418 to 0.490)	685.2 (343.8 to 1027)
Ds-EGFP non puncta	20.96 (13.70 to 44.64)	0.773 (0.659 to 0.888)	130.5 (-260.5 to 521.5)
Ds-EGFP *UAS-fbxl7* puncta	42.24 (31.77 to 63.00)	0.284 (0.252 to 0.315)	1088 (772.2 to 1403)
Ft-EGFP *UAS-fbxl7* non puncta	39.99 (30.28 to 58.89)	0.673 (0.599 to 0.747)	144.7 (20.85 to 268.5)
**Figure 7H**			
No induction 18°C	27.76 (19.02 to 51.35)	0.654 (0.57 to 0.741)	215.9 (-86.30 to 518.2)
1 hr induction	31.53 (24.16 to 45.36)	0.594 (0.543 to 0.646)	292.0 (149.1 to 434.9)
2 hr induction	29.17 (23.38 to 38.76)	0.668 (0.619 to 0.717)	314.2 (151.4 to 477.0)
4 hr induction	27.89 (21.08 to 41.23)	0.445 (0.403 to 0.486)	433.0 (129.7 to 736.3)
6 hr induction	19.55 (14.12 to 31.78)	0.381 (0.343 to 0.420)	610.3 (402.9 to 817.7)
24 hr induction	35.06 (25.92 to 54.14)	0.246 (0.219 to 0.273)	1280 (-233.2 to 2793)
No induction 29°C	26.27 (18.48 to 45.38)	0.645 (0.573 to 0.717)	358.0 (53.28 to 662.7)
